# Reinterpreting Dependency Schemes: Soundness Meets Incompleteness in DQBF

**DOI:** 10.1007/s10817-018-9482-4

**Published:** 2018-09-24

**Authors:** Olaf Beyersdorff, Joshua Blinkhorn, Leroy Chew, Renate Schmidt, Martin Suda

**Affiliations:** 1 School of Computing, University of Leeds, Leeds, UK; 2 School of Computer Science, University of Manchester, Manchester, UK; 3 Institute of Information Systems, Vienna University of Technology, Vienna, Austria

**Keywords:** Quantified Boolean formulas, DQBF, Dependency schemes, F.2.2 Nonnumerical algorithms and problems

## Abstract

Dependency quantified Boolean formulas (DQBF) and QBF dependency schemes have been treated separately in the literature, even though both treatments extend QBF by replacing the linear order of the quantifier prefix with a partial order. We propose to merge the two, by reinterpreting a dependency scheme as a mapping from QBF into DQBF. Our approach offers a fresh insight on the nature of soundness in proof systems for QBF with dependency schemes, in which a natural property called ‘full exhibition’ is central. We apply our approach to QBF proof systems from two distinct paradigms, termed ‘universal reduction’ and ‘universal expansion’. We show that full exhibition is sufficient (but not necessary) for soundness in universal reduction systems for QBF with dependency schemes, whereas for expansion systems the same property characterises soundness exactly. We prove our results by investigating DQBF proof systems, and then employing our reinterpretation of dependency schemes. Finally, we show that the reflexive resolution path dependency scheme is fully exhibited, thereby proving a conjecture of Slivovsky.

## Introduction

Research in automated reasoning has led to significant advances in the efficient solution of computationally hard problems [41]. SAT is the canonical ${{\mathbf {\mathsf{{NP}}}}}$-complete decision problem [14], yet state-of-the-art solvers using *conflict-driven clause learning* (CDCL) [5, 34] routinely solve instances with millions of variables [41]. Any decision procedure implicitly defines a *proof system* [15] for the input language, and proof-theoretic techniques can therefore be used to evaluate concrete implementations. CDCL, for example, is built on *resolution*, a well-studied propositional proof system on which there is a significant volume of research. This means that the trace of a CDCL solver on an unsatisfiable instance can be interpreted as a resolution proof of unsatisfiability.

SAT implementations serve a host of applications in computer science [10], in which they may be employed as ${{\mathbf {\mathsf{{NP}}}}}$ oracles in decision procedures for even harder problems [28]. One such problem is the decision problem for *quantified Boolean formulas* (QBF), the canonical ${{\mathbf {\mathsf{{PSPACE}}}}}$-complete language [38]. QBF extends propositional logic with existential and universal quantification over Boolean variables. Problem instances are typically written in prenex normal form, where variables are quantified in a linear order preceding the proposition.

Quantification presents a challenge for practitioners, as the quantifier prefix imposes *dependencies* between oppositely-quantified variables; for example, the chosen value of an existential *x* may depend upon that of every universal *u* that occurs earlier in the prefix. Put another way, a *model* for a QBF (the analogue of a satisfying assignment for a propositional formula) is a set of functions $\{f_x\}$ indexed over the existential variables, where $f_x$ assigns a truth value to every set of truth values for the universals preceding *x*.

This restricts the allowable order of variable assignments during search, which negatively impacts decision heuristics [26]. It is has been shown that many of these dependencies are ‘spurious’, in the sense that a model for the QBF exists in which the value of *x* does not change with respect to *u* [31], i.e. the truth value for *u* is absent from the input to $f_x$. In such cases the solver may assign variables more freely, improving the role of decision heuristics in search [26]. This has given rise to ‘dependency-aware’ QBF solving [27] based on *dependency schemes* [31], efficient algorithms that attempt to identify spurious dependencies.

**Fig. 1:**
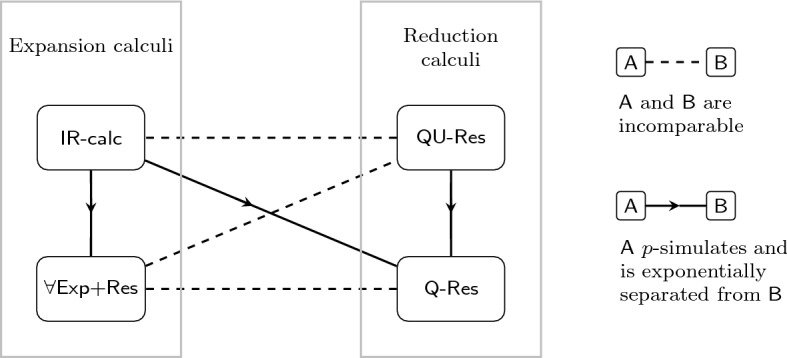
The simulation order of the four QBF proof systems considered in this paper

There are a host of QBF proof systems in the literature [4, 8, 37, 39], many of which have associated implementations [23, 27, 39]. In this paper, we focus on the four QBF systems depicted in Fig. 1 and investigate how the addition of a dependency scheme affects the soundness of the system. On the expansion side, the fundamental calculus $$\forall $$$$\textsf {Exp+Res}$$ [23] uses annotations to represent duplicate existential variables in the full expansion of a QBF (in which all universal variables have been eliminated), and is otherwise identical to propositional resolution. The stronger system $$\textsf {IR-calc}$$ [7], inspired by resolution in first-order logic, uses an additional instantiation rule to model ‘partial’ expansions. On the reduction side, the base system $$\textsf {Q-Res}$$ [24] employs resolution over existential variables in addition to a $$\forall $$-reduction rule that allows universal variables to be removed from clauses under certain conditions. $$\textsf {QU-Res}$$ [19] is a stronger calculus that extends $$\textsf {Q-Res}$$ with resolution over universal pivots. All four systems are sound and complete for the language of false QBFs (i.e., they are refutational calculi).

Our work establishes a relationship between QBF dependency schemes and *dependency quantified Booelean formulas* (DQBF) [21]. DQBF is a superset of QBF in which the linear order of the prefix is supplanted by Henkin quantifiers [21]. This allows arbitrary variable dependencies to be given explicitly using *support sets*, and results in a more expressive language that is ${{\mathbf {\mathsf{{NEXP}}}}}$-complete [1]. Complexity has not deterred researchers, and there is already a body of work on DQBF theory [13, 42], solving [17, 20], applications [1, 12, 20] and associated proof systems [3, 29].

### Our Contributions

DQBF and QBF dependency schemes have largely been treated separately in the literature; we contend that they are in fact very closely related. The key insight is that a dependency scheme, by identifying and removing spurious variable dependencies, is effectively replacing the linear order of quantification with a partial order. A DQBF, which specifies the allowable dependencies for variables with support sets, is capable of expressing such a partial order. For that reason, we propose to *redefine a dependency scheme as a mapping from QBF into DQBF*, in which the spurious dependencies identified by the scheme are left out of the support sets of the image formula.

Adopting this DQBF-centric approach yields benefits that surpass mere notational convenience. For example, a dependency scheme property known as *full exhibition*^1^ [6, 35] can be defined succinctly as follows: $\mathcal {D}$ is fully exhibited if and only if it maps every true QBF to a true DQBF. Second, new QBF calculi incorporating dependency schemes can be defined (and existing ones redefined) simply as the fragment of a corresponding DQBF system operating on the image formulas of the scheme. That is, given a DQBF calculus $\textsf {DQBF-P}$, we can define a class of QBF systems $\textsf {P}$($\mathcal {D}$) (parametrised by dependency scheme $\mathcal {D}$) as follows: for a fixed $\mathcal {D}$, $\textsf {P}$($\mathcal {D}$) is the set of $\textsf {DQBF-P}$ derivations from the image of $\mathcal {D}$ (a set of DQBFs). This allows us to translate results on soundness—and even incompleteness—from $\textsf {DQBF-P}$ to $\textsf {P}$($\mathcal {D}$), where $\textsf {P}$ is any of the systems in Fig. 1.

We summarise our results below. Part of this article is based on preliminary results reported in the conference proceedings [6, 9], which are here further elaborated and extended.

*New Results for Reduction Systems* We first generalise some existing results in the literature for $\textsf {DQBF-Q-Res}$ and $\textsf {Q}(\mathcal {D})\textsf {-Res}$ to the stronger systems $\textsf {DQBF-QU-Res}$ and $\textsf {QU}(\mathcal {D})\textsf {-Res}$. We first show that $\textsf {DQBF-QU-Res}$ is sound; under our new interpretation this implies that $\textsf {QU}(\mathcal {D})\textsf {-Res}$ is sound if $\mathcal {D}$ is fully exhibited. We also modify the example formula from [3] to show that $\textsf {DQBF-QU-Res}$ is incomplete. Moreover, we use the modified formula to construct a dependency scheme $\mathcal {D}$ that is not fully exhibited, but for which $\textsf {QU}(\mathcal {D})\textsf {-Res}$ is sound, thereby proving that full exhibition is not a necessary condition for soundness in the reduction systems parametrised by dependency scheme. Proceeding from the single example, we show that incompleteness in $\textsf {DQBF-QU-Res}$ is closely related to soundness in $\textsf {QU}(\mathcal {D})\textsf {-Res}$. Formally, we prove that the false DQBFs that cannot be refuted in $\textsf {DQBF-QU-Res}$ are precisely those formulas that are the image of a true QBF in some $\mathcal {D}$ for which $\textsf {QU}(\mathcal {D})\textsf {-Res}$ is sound.

*New Sound and Complete Expansion Systems* We lift the expansion-based QBF systems $$\forall $$$$\textsf {Exp+Res}$$ and $$\textsf {IR-calc}$$ to DQBF in the natural way, naming the new systems $$\textsf {DQBF-}$$$$\forall $$$$\textsf {Exp+Res}$$ and $$\textsf {DQBF-IR-calc}$$. Utilising a known transformation to the $$\mathsf {EPR}$$ fragment of first-order logic, we prove that both calculi are sound and complete for DQBF. We propose new expansion-based QBF systems $$\forall $$$$\textsf {Exp}(\mathcal {D})\textsf {+Res}$$ and $$\textsf {IR}(\mathcal {D})\textsf {-calc}$$ (parametrised by dependency scheme), defined conveniently in terms of their DQBF counterparts. Thanks to the first-order translation exploited at the DQBF level, we prove that each system is sound if and only if $$\mathcal {D}$$ is fully exhibited. We thereby provide a solid theoretical model for dependency schemes in expansion-based solving.

*Full Exhibition of*$\mathcal {D}^{\textsf {rrs}}$ In the penultimate section of the paper, we show that the reflexive resolution path dependency scheme $\mathcal {D}^{\textsf {rrs}}$ is fully exhibited, thus proving Slivovsky’s conjecture [35]. This is a welcome result for two reasons. Firstly, $\mathcal {D}^{\textsf {rrs}}$ is arguably the most important scheme in the literature, being the strongest known $\mathcal {D}$ for which $\textsf {Q}(\mathcal {D})\textsf {-Res}$ is sound. Secondly, in tandem with our other results this implies that $\textsf {P}(\mathcal {D}^{\textsf {rrs}})$ is sound, where $\textsf {P}$ is any of the four systems in Fig. 1. In particular, this promotes the use of the scheme in expansion-based solving, and therefore supports proposed future directions for QBF practice [22].

### Related Work

A host of dependency schemes have been proposed in the literature, including the standard ($\mathcal {D}^{\textsf {std}}$) [31] and reflexive resolution path ($\mathcal {D}^{\textsf {rrs}}$) [37] dependency schemes. Early experiments with the dependency-aware QCDCL solver DepQBF [27] demonstrated improved performance on certain benchmark sets [26]. A solid theoretical model for dependency-aware solving did not appear until later, in the shape of the calculus $\textsf {Q}(\mathcal {D})\textsf {-Res}$ [37], the parameterisation of $\textsf {Q-Res}$ by the dependency scheme $\mathcal {D}$. Dependency-awareness has not yet been implemented in expansion-based solvers, but it has been cited by the authors of RAReQS as a direction for future development [22].

The proposal of $\textsf {Q}(\mathcal {D})\textsf {-Res}$ raised some unexpected issues regarding soundness in dependency-aware solving. Perhaps most surprisingly, the so-called ‘optimal’ dependency scheme $\mathcal {D}^{\textsf {opt}}$ (conceived as the ideal scheme for QCDCL [26]) was shown to be unsuitable—$\textsf {Q}(\mathcal {D}^{\textsf {opt}})\textsf {-Res}$ is not sound [37]. Thus, as the soundness of $\textsf {Q}(\mathcal {D})\textsf {-Res}$ is not guaranteed, the question arose of how to determine whether or not the calculus is sound for a given scheme $\mathcal {D}$. In the absence of general methods, complicated ad hoc proofs of soundness (such as that for $\textsf {Q}(\mathcal {D}^{\textsf {rrs}})\textsf {-Res}$ [37]) have been published. A more elegant method was used by Slivovsky [35] to show the soundness of $\textsf {Q}(\mathcal {D}^{\textsf {std}})\textsf {-Res}$, using *full exhibition*. The proof proceeds by showing that (1) $\textsf {Q}(\mathcal {D})\textsf {-Res}$ is sound if $\mathcal {D}$ is fully exhibited and (2) $\mathcal {D}^{\textsf {std}}$ is fully exhibited. Slivovsky conjectured that $\mathcal {D}^{\textsf {rrs}}$ is also fully exhibited [35], leaving the proof as an open problem.

### Organisation of the Paper

After providing the necessary background in Sect. 2, we introduce the DQBF redefinition of dependency scheme in Sect. 3. Reduction and expansion systems are the focus of Sects. 4 and 5, respectively. In Sect. 6, we prove that $\mathcal {D}^{\textsf {rrs}}$ is fully exhibited. We offer some conclusions and further discussion in Sect. 7.

## Preliminaries

*Dependency Duantified Boolean Formulas* A *dependency quantified Boolean formula* (DQBF) is a formula over Boolean variables in which dependencies are given explicitly in the quantifier prefix. We deal exclusively with *prenex Skolem-form* DQBFs of the form $\varPhi = {\mathcal {Q}}\cdot {\phi }$, where:

- $\mathcal {Q} = \forall u_1 \cdots \forall u_m \exists x_1(S_1) \cdots \exists x_n(S_n)$ is the *quantifier prefix* in which all variables of $\phi$ are quantified either existentially or universally, and each existential variable $x_i$ is associated with a *support set*$S_i \subseteq \{u_1,\ldots ,u_m\}$ of universal variables.
- $\phi$ is a *conjunctive normal form* (CNF) matrix consisting of a conjunction of clauses, each of which is a disjunction of literals, and a literal is either a variable or its negation.

A quantified Boolean formula (QBF) is a DQBF in which the support sets are nested.^2^ That is, $\varPhi$ is a QBF if and only if $S_i \subseteq S_j$ for each $i,j \in [n]$ with $i < j$. We write $\mathsf {DQBF}$ and $\mathsf {QBF}$ to denote the set of all DQBFs and all QBFs respectively.

For a literal *l*, we write $\text{ var }(l) = z$ iff $l = \lnot z$ or $l = z$. For a clause *C*, we write $\text{ vars }(C) = \{\text{ var }(l) \mid l \in C\}$. For a DQBF $\varPhi$, $\text{ vars }(\varPhi )$ denotes the variables appearing in the quantifier prefix. We also use $C_\exists$ (respectively $C_\forall$) to denote the set of existential (respectively universal) literals in *C*. The empty clause is denoted by $\bot$.

An assignment to a set $V \subseteq \text{ vars }(\varPhi )$ is a function $\alpha :V \rightarrow \{0,1\}$ mapping variables to truth values, conventionally represented as a set of literals in which the literal $\lnot v$ (respectively *v*) represents the assignment $v \mapsto 0$ (respectively $v \mapsto 1$) for any $v \in V$. To simplify the presentation, we treat assignments interchangeably as either functions or as sets of literals, and remind the reader of the dual view wherever appropriate.

*DQBF Models* Given a DQBF $\varPhi = {\forall u_1 \cdots \forall u_m \exists x_1(S_1) \cdots \exists x_n(S_n)}\cdot {\phi }$, a *Skolem-function model* (or *model* for short) for $\varPhi$ is a set of functions $F = \{f_1, \dots ,f_n\}$ satisfying the following conditions:

- for each $i \in [n]$, $f_i$ is a function whose domain is the set of total assignments to $S_i$, that maps each such assignment to a truth value.
- The propositional formula $\phi ^\prime$, obtained by substituting each existential variable $x_i$ with (a propositional representation of) its Skolem function $f_i$, is a tautology.

A DQBF is true if and only if it has a model. The model *F* itself can be represented as a function mapping total assignments to the universals $\{u_1, \dots ,u_m\}$ (typically denoted $\alpha$) to total assignments to the existentials $\{x_1, \dots ,x_m\}$, in which $F(\alpha )$ is the assignment mapping each $x_i$ to $f_i(\alpha _i)$, where $\alpha _i$ is the restriction of $\alpha$ to the support set $S_i$. In this light, condition (b) above can be restated as ‘for every total assignment to the universals $\alpha$ the matrix $\phi$ is true under the joint assignment $\alpha \cup F(\alpha )$’.

**Fig. 2:**
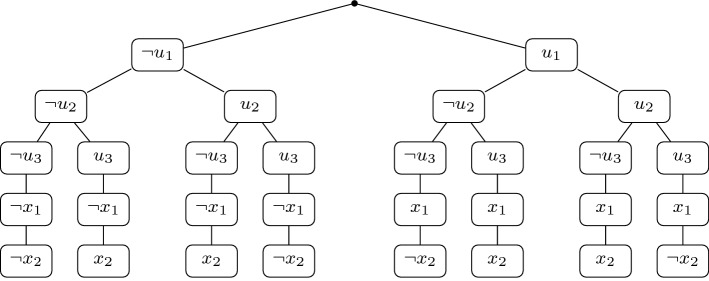
An assignment tree *T* for a DQBF $\varPhi = {\forall u_1 \forall u_2 \forall u_3 \exists x_1(u_1) \exists x_2 (u_2,u_3)}\cdot {\phi }$, whose matrix $\phi$ is satisfied if and only if $x_1 = u_1$ and $x_2 = u_2 \oplus u_3$. *T* represents a model for $\varPhi$, since it implicitly defines the unique Skolem functions that satisfy the matrix

For some technical portions of the paper, we prefer an equivalent formulation of DQBF semantics, based on an adaptation of QBF *assignment trees* [32]. The nesting of support sets in a QBF prefix allows a model to be depicted naturally as a tree, with branching over universal variables. A DQBF model *F* can also be depicted as a tree, in which branching on all universals takes place consecutively from the root, as shown in Fig. 2. For each $\alpha$ in the domain of *F*, the literals of the set $\alpha \cup F(\alpha )$ are written (in the order they appear in the prefix, i.e., all universals first) on a unique path from the root of the tree to some leaf. As such, *F* can be uniquely identified with a set of $2^m$*paths*, each of which is one of the sets $\alpha \cup F(\alpha )$. An arbitrary assignment tree *T* for $\varPhi$ represents a model if and only if it implicitly defines a set of Skolem functions. This proves to be a convenient interpretation specifically for the detailed proofs in Sect. 6. We therefore postpone further details on assignment trees to that section.

*Proof SystemsResolution* is a well-studied refutational proof system for propositional CNF formulas with a single inference rule: the *resolvent*$C_1 \cup C_2$ may be derived from clauses $C_1 \cup \{x\}$ and $C_2 \cup \{\lnot x\}$, where variable *x* is called the *pivot*. Resolution is sound and *refutationally* complete: that is, the empty clause $\bot$ can be derived from a CNF if and only if it is unsatisfiable. All the proof systems for DQBF (and QBF) considered in this paper are refutational calculi (i.e., they prove falsity) based on resolution.

*Q-resolution* ($\textsf {Q-Res}$) [24] is the standard refutational calculus for prenex QBF. In addition to resolution over existential pivots, the calculus has a *universal reduction rule* which allows a clause *C* to be derived from $C \cup \{l\}$, provided $\text{ var }(l)$ is a universal variable absent from the support set of each existential variable appearing in *C*. Tautologies are explicitly forbidden; one may not derive a clause containing both *z* and $\lnot z$. $\textsf {QU-Res}$ [19] is the extension of $\textsf {Q-Res}$ that allows resolution over universal pivots. We omit their formal definitions, since (on the set of QBFs) each coincides with the corresponding DQBF system, whose definition appears in the main text.

For a DQBF resolution system $\textsf {P}$, a $\textsf {P}$-*derivation* of a clause *C* from a PCNF $\varPhi$ is a sequence $C_1, \dots , C_k$ of clauses in which $C=C_k$, and each clause is either an axiom or is derived from previous clauses in the sequence using an inference rule. A *refutation* of $\varPhi$ is a derivation of the empty clause $\bot$ from $\varPhi$.

A proof system $\mathsf {P}$*p-simulates* a system $\mathsf {Q}$ (denoted $\mathsf {Q} \le _p \mathsf {P}$) if each $\mathsf {Q}$-proof can be transformed in polynomial time into a $\mathsf {P}$-proof of the same formula [15]. The systems $\mathsf {P}$ and $\mathsf {Q}$ are *p-equivalent* (denoted $\mathsf {P}\equiv _p \mathsf {Q}$) if $\mathsf {Q}\le _p \mathsf {P}$ and $\mathsf {P}\le _p \mathsf {Q}$.

## A DQBF Interpretation of Dependency Schemes

In this section, we recall the traditional interpretation of dependency schemes, before presenting our new interpretation based on DQBF.

### The Traditional Interpretation of Dependency Schemes

The traditional interpretation of dependency schemes originates from [30]. That model was soon modified to work with binary relations [31], and was subsequently employed by a number of authors, e.g. [6, 26, 36]. We give a high level description, for comparison with the DQBF interpretation proposed in the next subsection.

For an arbitrary QBF $\varPhi$, the trivial dependency relation $\mathcal {D}^{\textsf {trv}} (\varPhi )$ is the set of pairs $(z_1,z_2) \in \text{ vars }(\varPhi ) \times \text{ vars }(\varPhi )$ for which (1) $z_1$ and $z_2$ are oppositely quantified, and (2) $z_1$ is quantified before $z_2$ when the quantifier prefix is arranged in the natural linear order (cf. [31]). (Using our DQBF notation, condition (2) means that $z_1$ is in the support set of $z_2$ if the latter is existential, otherwise $z_2$ is not in the support set of $z_1$.) From there, a dependency scheme $\mathcal {D}$ is a function that maps each QBF to a binary relation, and satisfies $\mathcal {D} (\varPhi ) \subseteq \mathcal {D}^{\textsf {trv}} (\varPhi )$ for each QBF $\varPhi$.^3^ The binary relation identifies sets of pairs $(z_1,z_2)$ for which $z_2$ is considered dependent on $z_1$. Accordingly, the existence of a pair $(z_1,z_2) \in \mathcal {D} (\varPhi )$ should be interpreted as ‘$z_2$ depends on $z_1$ in $\varPhi$ according to $\mathcal {D}$ ’. Pairs not included in the binary relation represent independencies; that is, a pair $(z_1,z_2) \in \mathcal {D}^{\textsf {trv}} (\varPhi ) {\setminus } \mathcal {D} (\varPhi )$ should be interpreted as ‘$z_2$ is independent of $z_1$ in $\varPhi$ according to $\mathcal {D}$ ’.

An important point for the present context is that we may restrict our attention to the (in)dependence of existentials on universals. The dual notion of (in)dependence of universals on existentials, which is equally important in practice, does not feature in theoretical models of solving. This is a quite convenient state of affairs, afforded by the fact that theorists deal with *refutational* calculi.

### Redefining Dependency Schemes in Terms of DQBF

The principal idea is that the binary relation $\mathcal {D} (\varPhi )$ can be written as a DQBF quantifier prefix. This proposal seems perfectly natural. In practice, the primary purpose for a dependency scheme is to replace the linear order of a QBF prefix with a partial order that more accurately reflects the dependency structure of the instance. Note that a DQBF prefix, in general, represents a partial order. In other words, a dependency scheme can be viewed as a transformation that maps a QBF to a suitable DQBF (with the same matrix), whose quantifier prefix expresses dependencies according to the scheme. As we consider only existential dependencies in our theoretical models, we can work exclusively with Skolem-form DQBFs.

Since dependency schemes are in the business of identifying non-trivial independencies, we inevitably want to map QBFs to DQBFs in which the support sets are smaller (whereas the variable sets and the matrix should not change). We define a *strength* relation to capture this notion.

#### Definition 1

(strength) Let $\varPhi := {\forall u_1 \cdots \forall u_m \exists x_1(S_1) \cdots \exists x_n(S_n)}\cdot {\phi }$. A DQBF $\varPhi ^\prime$ is *stronger than* $\varPhi$ if and only if $\varPhi ^\prime = {\forall u_1 \cdots \forall u_m \exists x_1(S^\prime _1) \cdots \exists x_n(S^\prime _n)}\cdot {\phi }$ and $S^\prime _i \subseteq S_i$ for each $i \in [n]$.

A dependency scheme may now be naturally (and concisely) defined as a function mapping QBFs to stronger DQBFs.

#### Definition 2

(dependency scheme) A *dependency scheme* is a function $\mathcal {D}:\mathsf {QBF} \rightarrow \mathsf {DQBF}$ that maps each QBF $\varPhi$ to a stronger DQBF $\mathcal {D} (\varPhi )$.

The DQBF interpretation also facilitates an illuminating definition of an important property called *full exhibition* [6, 35]. Full exhibition will feature prominently in this work, in which we demonstrate the greater potential of the concept in connection with the soundness of dependency systems.

According to previous work [6, 35], a dependency scheme $\mathcal {D}$ is said to be fully exhibited if and only if the following property holds for each true QBF $\varPhi$: there exists a particular model for $\varPhi$ in which, for each existential *x* and universal *u* in $\text{ vars }(\varPhi )$, *x* does not depend on *u* whenever *x* is independent of *u* in $\varPhi$ according to $\mathcal {D}$. This is precisely the same as saying that the associated DQBF $\mathcal {D} (\varPhi )$ is true. Hence we considerably simplify the definition of the concept.

#### Definition 3

(full exhibition) A dependency scheme $\mathcal {D}$ is *fully exhibited* if and only if it maps each true QBF to a true DQBF.

Note that a false QBF is always mapped to a false DQBF by any dependency scheme, due to the strength condition.

We conclude this section by showing that the pairwise comparison of schemes also fits neatly into our reinterpretation. Schemes are compared by the notion of generality, whereby one scheme is considered more general than another if it is capable of identifying more non-trivial independencies.

#### Definition 4

(generality) Let $\mathcal {D}$ and $\mathcal {D} ^\prime$ be dependency schemes. We say that $\mathcal {D}$ is *at least as general as*$\mathcal {D} ^\prime$ if and only if $\mathcal {D} (\varPhi )$ is stronger than $\mathcal {D} ^\prime (\varPhi )$ for each QBF $\varPhi$.

## Variable Dependencies in $\textsf {Q-Res}$ and $\textsf {QU-Res}$

### DQBF Systems

Balabanov et al. [3] introduced the DQBF calculus $\textsf {DQBF-Q-Res}$ as the natural generalisation of the QBF system $\textsf {Q-Res}$. The salient feature of $\textsf {DQBF-Q-Res}$ is that $\forall$-reduction of a universal literal *l* from a clause *C* is allowed if and only if $\text{ var }(l)$ does not appear in the support set of any existential variable appearing in *C*. (Note that $\textsf {Q-Res}$ and $\textsf {DQBF-Q-Res}$ are equivalent on QBFs.)

**Fig. 3:**
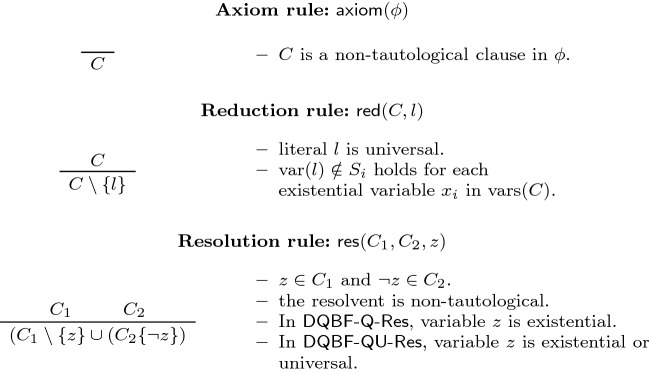
The rules of the proof systems $\textsf {DQBF-Q-Res}$ [3] and $\textsf {DQBF-QU-Res}$. The input DQBF is denoted $\varPhi := {\forall u_1\cdots \forall u_m \exists x_1(S_1)\cdots \exists x_n(S_n)}\cdot {\phi }$

The calculus is easily extended to include resolution over universal pivots. We call the resulting system $\textsf {DQBF-QU-Res}$, as it is the natural generalisation of $\textsf {QU-Res}$ to DQBF. The formal description of both systems is given in Fig. 3. Balabanov et al. [3] went on to show that $\textsf {DQBF-Q-Res}$ is sound, yet incomplete, for DQBF. In this subsection, we show that both results carry over to $\textsf {DQBF-QU-Res}$.

Inspection of the original proof of soundness for $\textsf {DQBF-Q-Res}$ [3, Theorem 5] reveals that the argument does not rely on the existential quantification of a pivot variable, and therefore lifts to $\textsf {DQBF-QU-Res}$. Nonetheless, we provide a complete proof of the result, keeping the present work self-contained. Moreover, our proof method (which differs from [3]) is better suited to the context of this paper.

#### Theorem 1

The DQBF calculus $\textsf {DQBF-QU-Res}$ is sound.

#### Proof

We show that there is no $\textsf {DQBF-QU-Res}$ refutation of a true DQBF. Aiming for contradiction, let $\varPhi = {\mathcal {Q}}\cdot {\phi }$ be a true DQBF and suppose that $\pi = C_1,\ldots ,C_k$ is a $\textsf {DQBF-QU-Res}$ refutation of $\varPhi$. Further, for each $i \in [k]$, let $\phi _i = \{C_1, \dots ,C_i\}$.

Now, let *F* be any model for $\varPhi$. We prove by induction on $i \in [k]$ that *F* is a model for ${\mathcal {Q}}\cdot {\phi _i}$. Hence at step $i=k$, we deduce that $\varPhi = {\mathcal {Q}}\cdot {\phi _k}$ is true, a clear contradiction since $\phi _k$ contains the empty clause $C_k =\bot$.

The base case $i=1$ is established trivially, since $C_1$ must be an axiom clause from $\phi$. For the inductive step, let $1 < i \le k$, suppose that *F* is a model for ${\mathcal {Q}}\cdot {\phi _{i-1}}$, and let $\alpha$ be a total assignment to the universal variables of $\varPhi$. The case where $C_i$ is an axiom is identical to the base case, hence we assume that $C_i$ is derived either by resolution or by $\forall$-reduction. In either case we show that $\alpha \cup F(\alpha )$ satisfies $C_i$, and hence *F* is a model for ${\mathcal {Q}}\cdot {\phi _{i-1} \cup \{C_i\}} = {\mathcal {Q}}\cdot {\phi _i}$, completing the inductive step.

1. Suppose that $C_i$ was derived by resolution from clauses $C_a$ and $C_b$. Then, since $C_a,C_b \in \phi _{i-1}$, $\alpha \cup F(\alpha )$ satisfies both clauses by the inductive hypothesis, and hence satisfies $C_i$ by the logical correctness of propositional resolution.
2. Suppose, on the other hand, that $C_i$ was derived by $\forall$-reduction from some clause $C_i \cup \{l\}$, and assume w.l.o.g. that $l \in \alpha$. Also, let $\alpha ^\prime$ be the assignment that agrees with $\alpha$ on every universal variable except $\text{ var }(l)$; that is, define $\alpha ^\prime := (\alpha \cup \{\lnot l\}) {\setminus } \{l\}$. Since $\lnot l \in \alpha ^\prime$, and $\alpha ^\prime \cup F(\alpha ^\prime )$ satisfies $C_i \cup \{l\}$ by the inductive hypothesis, $\alpha ^\prime \cup F(\alpha ^\prime )$ satisfies $C_i$. By definition of $\forall$-reduction, $\text{ var }(l) = u$ is a universal variable satisfying $u \notin S$ for each support set *S* of an existential in $\text{ vars }(C_i)$. It follows that $F(\alpha )$ and $F(\alpha ^\prime )$ agree on all existential variables in $C_i$. Also, $\alpha$ and $\alpha ^\prime$ agree on all universal variables except *u*. Noting that $\lnot l\notin C_i$ (since $C_i \cup \{l\}$ is not a tautology), variable *u* does not appear in $C_i$, and it follows that $\alpha \cup F(\alpha )$ and $\alpha ^\prime \cup F(\alpha ^\prime )$ agree on $\text{ vars }(C_i)$. Therefore $\alpha \cup F(\alpha )$ satisfies $C_i$.

$$\square $$

We conclude this subsection by showing that $\textsf {DQBF-QU-Res}$ is incomplete. Balabanov et al. [3] gave the following false DQBF that cannot be refuted in $\textsf {DQBF-Q-Res}$.

#### Example 1

Let $\varPsi$ be the DQBF with prefix

$$\begin{aligned} \forall u_1 \forall u_2 \exists x_1 (u_1) \exists x_2 (u_2) \end{aligned}$$

and matrix consisting of the clauses

$$\begin{aligned} \begin{array}{ll} \{x_1,x_2,u_1\}, &{} \qquad \qquad \{\lnot x_1,\lnot x_2,u_1\},\\ \{x_1, x_2,\lnot u_1,\lnot u_2\},&{} \qquad \qquad \{\lnot x_1, \lnot x_2,\lnot u_1,\lnot u_2\},\\ \{x_1,\lnot x_2,\lnot u_1,u_2\}, &{} \qquad \qquad \{\lnot x_1, x_2,\lnot u_1,u_2\}.\\ \end{array} \end{aligned}$$

In the following example, we modify the above formula $\varPsi$ by doubling universal variables, a useful technique introduced by Balabanov et al. [4]. The modified formula $\varPsi ^\prime$ remains false, while the addition of duplicate variables ensures that no resolution steps over universal pivots are possible.

#### Example 2

Let $\varPsi ^\prime$ be the DQBF with prefix

$$\begin{aligned} \forall u_1 \forall u^\prime _1 \forall u_2 \forall u^\prime _2 \exists x_1 (u_1,u^\prime _1) \exists x_2 (u_2,u^\prime _2) \end{aligned}$$

and matrix consisting of the clauses

$$\begin{aligned} \begin{array}{ll} \{x_1,x_2,u_1,u^\prime _1\}, &{} \qquad \qquad \{\lnot x_1,\lnot x_2,u_1,u^\prime _1\},\\ \{x_1, x_2,\lnot u_1, \lnot u^\prime _1,\lnot u_2, \lnot u^\prime _2\},&{}\qquad \qquad \{\lnot x_1, \lnot x_2,\lnot u_1,\lnot u^\prime _1,\lnot u_2,\lnot u^\prime _2\},\\ \{x_1,\lnot x_2,\lnot u_1, \lnot u^\prime _1,u_2, u^\prime _2\}, &{} \qquad \qquad \{\lnot x_1, x_2,\lnot u_1,\lnot u^\prime _1,u_2,u^\prime _2\}.\\ \end{array} \end{aligned}$$

We first show that $\varPsi ^\prime$ is false. Note that, due to the doubling of universal variables, any model $F^\prime$ for $\varPsi ^\prime$ gives rise to a model *F* for the original formula $\varPsi$, in which the duplicates $u_1^\prime$ and $u^\prime _2$ are removed. (This is achieved by restricting the domain of $F^\prime$ to those assignments in which $u_i$ and $u^\prime _i$ are assigned similarly for $i \in \{1,2\}$, and then ignoring the assignments to $u_1^\prime$ and $u^\prime _2$.) However, it was shown by Balabanov et al. [3] that no model for the original formula exists.

Now we show that there is no $\textsf {DQBF-QU-Res}$ refutation of $\varPhi$. In fact, no clauses whatsoever can be derived from the matrix of $\varPsi ^\prime$. This is easy to verify as follows. Any resolution step produces a tautological resolvent, which is explicitly forbidden. No $\forall$-reduction step is possible, because every clause contains both existential variables $x_1$ and $x_2$, and the union of support sets $S_1 \cup S_2$ contains all the universal variables of $\varPhi$.

### Systems for QBF with Dependency Schemes

A major benefit of the DQBF interpretation is that QBF calculi augmented with dependency schemes can be defined as fragments of DQBF calculi. In this subsection, we expose the connection between $\textsf {DQBF-QU-Res}$ and the QBF calculus $\textsf {QU}(\mathcal {D})\textsf {-Res}$, the system that supplements $\textsf {Q}(\mathcal {D})\textsf {-Res}$ [37] with resolution over universal pivots. In addition to improved understanding, the connection fosters further proofs of soundness that are almost immediate.

Recalling our discussion of the traditional interpretation of dependency schemes in Sect. 3.1, we make the following observation: for a QBF $\varPhi$, the allowable inferences in $\textsf {Q}(\mathcal {D})\textsf {-Res}$ (as originally presented by Slivovsky and Szeider [37]) are exactly those inferences allowable in a $\textsf {DQBF-Q-Res}$ derivation from the DQBF $\mathcal {D} (\varPhi )$. This continues to hold in the presence of universal resolution.

Following this observation, it is indeed possible to formulate the definition of $\textsf {QU}(\mathcal {D})\textsf {-Res}$ neatly in terms of $\textsf {DQBF-QU-Res}$, since the former is essentially the restriction of the latter to the image of $\mathcal {D}$. To do this, we first define a general notion of *projection* that captures the condition by which a QBF proof system is a fragment of a DQBF system.

#### Definition 5

(projection) Let $\mathcal {D}$ be a dependency scheme and let $\textsf {Q}$ be a DQBF calculus. The *projection *$\textsf {P}$* of *$\textsf {Q}$* according to*$\mathcal {D}$ is the QBF calculus whose derivations from $\varPhi$ are exactly the $\textsf {Q}$-derivations from $\mathcal {D} (\varPhi )$, for each QBF $\varPhi$.

#### Definition 6

For each dependency scheme $\mathcal {D}$, the QBF calculus $\textsf {QU}(\mathcal {D})\textsf {-Res}$ is the projection of $\textsf {DQBF-QU-Res}$ according to $\mathcal {D}$.

The idea of defining QBF systems parametrised by dependency scheme as projections of DQBF systems is entirely consistent with existing literature, i.e. with the existing definition of $\textsf {Q}(\mathcal {D})\textsf {-Res}$.

#### Proposition 1

$\textsf {Q}(\mathcal {D})\textsf {-Res}$ is the projection of $\textsf {DQBF-Q-Res}$ according to $\mathcal {D}$, for each dependency scheme $\mathcal {D}$.

#### Proof

Let $\mathcal {D}$ be a dependency scheme. To verify the observation, we show that the three rules of inference in $\textsf {Q}(\mathcal {D})\textsf {-Res}$ (as presented in [35]) are exactly equivalent to the three rules of $\textsf {DQBF-Q-Res}$ when the input formula is a QBF $\varPhi := {\forall u_1 \cdots \forall u_m \exists x_1(S_1) \cdots \exists x_n(S_n)}\cdot {\phi }$. By $\mathcal {T} (\varPhi ) \subseteq \{(u_i,x_j) : u_i \in S_j\}$ we denote the traditional interpretation of the dependency scheme $\mathcal {D}$ on $\varPhi$ (as in Subsect. 3.1), and observe that $(u_i,x_j) \in \mathcal {T} (\varPhi ) \Leftrightarrow u_i \in S^\prime _j$, where $S^\prime _j$ is the support set for $x_j$ in $\mathcal {D} (\varPhi )$.

The ‘input clause’ and ‘resolution’ rules of $\textsf {Q}(\mathcal {D})\textsf {-Res}$ are exactly identical to the axiom and resolution rules in $\textsf {DQBF-Q-Res}$. The ‘$\forall (\mathcal {D})$-reduction’ rule allows a clause $C {\setminus } \{l\}$ to be inferred from *C* provided that $\text{ var }(l)$ is universal and $(\text{ var }(l),x_j) \notin \mathcal {T} (\varPhi )$ for each existential $x_j \in \text{ vars }(C)$. Since $(\text{ var }(l),x_j) \notin \mathcal {T} (\varPhi ) \Leftrightarrow \text{ var }(l) \notin S^\prime _j$, exactly the same inferences can be made by the reduction rule in $\textsf {DQBF-Q-Res}$. $\square$

The notion of projection, in combination with Theorem 1, fosters a straightforward proof of the following soundness theorem, which extends the analogous result for $\textsf {Q}(\mathcal {D})\textsf {-Res}$ [35].

#### Theorem 2

The QBF calculus $\textsf {QU}(\mathcal {D})\textsf {-Res}$ is sound if $\mathcal {D}$ is fully exhibited.

#### Proof

Let $\mathcal {D}$ be fully exhibited and let $\varPhi$ be a true QBF. Since $\mathcal {D} (\varPhi )$ is true (by definition of full exhibition), there is no $\textsf {DQBF-QU-Res}$ refutation of $\mathcal {D} (\varPhi )$, by Theorem 1. As $\textsf {QU}(\mathcal {D})\textsf {-Res}$ is the projection of $\textsf {DQBF-QU-Res}$ according to $\mathcal {D}$, there is no $\textsf {Q}(\mathcal {D})\textsf {-Res}$ refutation of $\varPhi$, so $\textsf {QU}(\mathcal {D})\textsf {-Res}$ is sound. $\square$

Note that $\textsf {QU}(\mathcal {D})\textsf {-Res}$ is complete regardless of the choice of $\mathcal {D}$, since it trivially *p*-simulates the complete system $\textsf {QU-Res}$.

Whereas full exhibition is sufficient for soundness in $\textsf {QU}(\mathcal {D})\textsf {-Res}$, it is not necessary; that is, full exhibition does not characterise soundness there. Considering Example 2, it is possible for a scheme $\mathcal {D}$ to map a true QBF to a false DQBF that cannot be refuted in $\textsf {DQBF-QU-Res}$. In such a situation, $\mathcal {D}$ is not fully exhibited, but $\textsf {QU}(\mathcal {D})\textsf {-Res}$ remains sound, precisely because the $\textsf {DQBF-QU-Res}$ fragment to which it corresponds is incomplete.

#### Proposition 2

There exists a dependency scheme $\mathcal {D}$ that is not fully exhibited for which $\textsf {QU}(\mathcal {D})\textsf {-Res}$ is sound.

#### Proof

Let $\varPsi ^\prime = {\mathcal {Q}}\cdot {\psi }$ be the DQBF from Example 2, and recall that the existential variables of $\varPsi ^\prime$ are $x_1$ and $x_2$ with corresponding support sets $S^\prime _1 = \{u_1,u^\prime _1\}$ and $S^\prime _2 = \{u_2,u_2^\prime \}$.

Now, let $\varPsi := {\forall u_1\forall u^\prime _1 \forall u_2\forall u^\prime _2 \exists x_1(S_1)\exists x_2(S_2)}\cdot {\psi }$ be the formula with support sets $S_1 := S_2 := \{u_1,u^\prime _1,u_2,u^\prime _2\}$. Observe that $\varPsi$ is a QBF and that $\varPsi ^\prime$ is stronger than $\varPsi$. Moreover, $\varPsi$ is a true QBF: For an assignment to the universal variables including $u_1 = 1$ and $u_2 = 0$, putting $x_1 = x_2$ satisfies all the clauses in $\psi$. In all other cases, putting $x_1 \ne x_2$ satisfies all clauses.

Now, we construct a dependency scheme $\mathcal {D}$ as follows. Let $\mathcal {D} (\varPsi ) = \varPsi ^\prime$, and let $\mathcal {D} (\varPhi ) = \varPhi$ for every other QBF $\varPhi \ne \varPsi$. Note that $\mathcal {D}$ is not fully exhibited, since it maps the true QBF $\varPsi$ to the false DQBF $\varPsi ^\prime$. Also note that $\textsf {QU}(\mathcal {D})\textsf {-Res}$ and $\textsf {QU-Res}$ are identical on all QBFs other than $\varPsi$.

We observe that $\textsf {QU}(\mathcal {D})\textsf {-Res}$ cannot refute $\varPsi$, as there is no $\textsf {DQBF-QU-Res}$ refutation of $\varPsi ^\prime$ (Example 2). Nor can $\textsf {QU}(\mathcal {D})\textsf {-Res}$ refute any other true QBF, by the soundness of $\textsf {QU-Res}$. Therefore $\textsf {QU}(\mathcal {D})\textsf {-Res}$ is sound. $\square$

The relationship between soundness and incompleteness, exploited in the preceding proof, allows us to equate two open problems. That is, if one determines the set of dependency schemes for which $\textsf {QU}(\mathcal {D})\textsf {-Res}$ is sound, then one determines the set of false DQBFs that $\textsf {DQBF-QU-Res}$ cannot refute. A characterisation of soundness in $\textsf {QU}(\mathcal {D})\textsf {-Res}$, therefore, also characterises incompleteness in $\textsf {DQBF-QU-Res}$, and vice versa. We formalise that notion exactly with the following theorem.

#### Theorem 3

Let *I* be the set of false DQBFs that have no $\textsf {DQBF-QU-Res}$ refutation, and let *S* be the set of dependency schemes $\mathcal {D}$ for which $\textsf {QU}(\mathcal {D})\textsf {-Res}$ is sound. Then *I* is the set of false DQBFs that are the image of a true QBF under some $\mathcal {D} \in S$.

#### Proof

If $\varPhi$ is true and $\textsf {QU}(\mathcal {D})\textsf {-Res}$ is sound, then $\mathcal {D} (\varPhi )$ has no $\textsf {DQBF-QU-Res}$ refutation (by definition of projection). Hence we only need show that each DQBF in *I* is the image of a true QBF under some dependency scheme in *S*.

Let $\varPhi = {\forall u_1 \cdots \forall u_m \exists x_1 (S_1) \cdots \exists x_n (S_n)}\cdot {\phi }$ be a DQBF in the set *I*, and consider the QBF $\varPhi ^\prime = {\forall u_1 \cdots \forall u_m \exists x_1 (S^\prime _1) \cdots \exists x_n (S^\prime _n)}\cdot {\phi }$ in which $S^\prime _i = \{u_1, \dots ,u_m\}$ for each $i \in [n]$. Note that $\varPhi$ is stronger than $\varPhi ^\prime$.

We first show that $\varPhi ^\prime$ is a true QBF. Aiming for contradiction, suppose that $\varPhi ^\prime$ is false. Then there exists a $\textsf {QU-Res}$ refutation $\pi$ of $\varPhi ^\prime$, by completeness of that calculus. In $\pi$, no universal is $\forall$-reduced from a clause containing an existential variable (as the support sets $S^\prime _i$ are maximal), and so $\pi$ is also a legal $\textsf {DQBF-QU-Res}$ refutation of $\varPhi$. However, no such refutation exists, by definition of *I*.

It remains to construct a dependency scheme $\mathcal {D}$, for which $\textsf {QU}(\mathcal {D})\textsf {-Res}$ is sound, satisfying $\mathcal {D} (\varPhi ^\prime ) = \varPhi$. That is achieved simply by putting $\mathcal {D} (\varPsi ) = \varPsi$ for all other QBFs $\varPsi \ne \varPhi ^\prime$. $\square$

Given the tight connection to DQBF incompleteness, the characterisation of soundness in $\textsf {QU}(\mathcal {D})\textsf {-Res}$ remains an interesting open problem. However, with Proposition 3 we argue that schemes that are not fully exhibited are largely unimportant from a proof complexity point of view. The use of a sound scheme that is not fully exhibited can always be *p*-simulated by the use of a fully exhibited one.^4^

#### Proposition 3

Let $$\mathcal {D} $$ be a dependency scheme for which $$\textsf {QU}(\mathcal {D})\textsf {-Res}$$ is sound. There exists a fully exhibited dependency scheme $$\mathcal {D} ^{\prime }$$, with $$\mathcal {D}$$ at least as general as $$\mathcal {D} ^\prime $$, for which $$\textsf {QU}(\mathcal {D})\textsf {-Res}$$$$\equiv _p$$$$\textsf {QU}(\mathcal {D} ^\prime )\textsf {-Res}$$.

#### Proof

We let $\mathcal {D} ^\prime$ be the dependency scheme defined as follows:

$$\begin{aligned} \mathcal {D} ^\prime (\varPhi ) = {\left\{ \begin{array}{ll} \mathcal {D} (\varPhi ) &{}\quad \text{ if } \varPhi \text{ is } \text{ false }\,,\\ \varPhi &{}\quad \text{ if } \varPhi \text{ is } \text{ true }\,. \end{array}\right. } \end{aligned}$$

It is clear that $\mathcal {D}$ is at least as general as $\mathcal {D} ^\prime$. $\mathcal {D} ^\prime$ is the identity transformation on true QBFs, and is therefore trivially fully exhibited. Since $\mathcal {D}$ and $\mathcal {D} ^\prime$ agree on all false QBFs, every $\textsf {QU}(\mathcal {D})\textsf {-Res}$ refutation is a $\textsf {QU}(\mathcal {D} ^\prime )\textsf {-Res}$ refutation, and vice versa. Hence $\textsf {QU}(\mathcal {D})\textsf {-Res}$ and $\textsf {QU}(\mathcal {D} ^\prime )\textsf {-Res}$ are *p*-equivalent. $\square$

## Dependencies in Expansion-Based Calculi

In this section, we apply our DQBF interpretation of dependency schemes to expansion-based proof systems. In Sect. 5.1, we lift the existing QBF calculi $$\forall $$$$\textsf {Exp+Res}$$ and $$\textsf {IR-calc}$$ to DQBF. Exploiting a natural translation to the $$\mathsf {EPR}$$ fragment of first-order logic, we prove that the resulting systems $$\textsf {DQBF-}$$$$\forall $$$$\textsf {Exp+Res}$$ and $$\textsf {DQBF-IR-calc}$$ remain sound and complete. Further, in Sect. 5.2 we introduce $$\forall $$$$\textsf {Exp}(\mathcal {D})\textsf {+Res}$$ and $$\textsf {IR}(\mathcal {D})\textsf {-calc}$$, the corresponding QBF systems parametrised by dependency scheme, and prove that full exhibition provides a complete characterisation for soundness in both systems.

### Two Sound and Complete Systems for DQBF

Let us first explain the concept of expansion with a simple example. Consider the DQBF

$$\begin{aligned} {\forall u \forall v \exists x(u) \exists y(v)}\cdot {\phi (u,v,x,y)}, \end{aligned}$$

and note that it is semantically equivalent to the DQBF

$$\begin{aligned} {\forall v \exists x^0()\exists x^1() \exists y(v)}\cdot {\phi (0,v,x^0,y) \wedge \phi (1,v,x^1,y)} \end{aligned}$$

in which we have ‘expanded’ the variable *u*. Since *x* depends on *u*, we introduce two distinct variables $x^0$ and $x^1$ in the expanded formula, one for each possible assignment to *u*, and give to each the support set of *x* with *u* removed (in this case yielding the empty support set). In contrast, the variable *y* does not include *u* in its support set, and therefore remains unchanged. A subsequent expansion of the remaining universal *v* yields the fully existentially quantified formula

$$\begin{aligned} {\exists x^0 \exists x^1 \exists y^0 \exists y^1}\cdot {\phi (0,0,x^0,y^0) \wedge \phi (0,1,x^0,y^1) \wedge \phi (1,0,x^1,y^0) \wedge \phi (1,1,x^1,y^1)} \end{aligned}$$

that is also semantically equivalent to the original DQBF.

It is convenient to annotate duplicate variables with the reason for their creation. For example, we will use $x^{\lnot u}$ instead of $x^0$ (where $\lnot u$ corresponds to the assignment $u \mapsto 0$), and likewise $x^{u}$ instead of $x^1$. Since expansion duplicates each dependent existential, the complete expansion of an arbitrary DQBF introduces $2^{|S|}$ copies of the variable with support set *S*, each of which is annotated with a complete assignment to *S*.

**Fig. 4:**
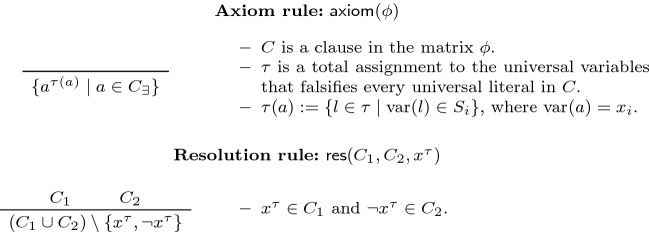
The rules of $$\textsf {DQBF-}$$$$\forall $$$$\textsf {Exp+Res}$$, where $$\varPhi = {\forall u_1\cdots \forall u_m \exists x_1(S_1)\cdots \exists x_n(S_n)}\cdot {\phi } $$ is the input DQBF

*Description of the Systems* Our base expansion calculus $$\textsf {DQBF-}$$$$\forall $$$$\textsf {Exp+Res}$$ works simply by applying propositional resolution to clauses in the complete expansion of a DQBF $$\varPhi $$. Accordingly, one may take any clause from the matrix and do the following: select a total assignment to universal variables that falsifies all the universal literals in the clause, and remove all universal literals while annotating the remaining existentials with the assignment. The resulting clause appears in the complete expansion of $$\varPhi $$, and may therefore be introduced as an axiom in a $$\textsf {DQBF-}$$$$\forall $$$$\textsf {Exp+Res}$$ derivation from $$\varPhi $$. The rules of $$\textsf {DQBF-}$$$$\forall $$$$\textsf {Exp+Res}$$ are given formally in Fig. 4.

**Fig. 5:**
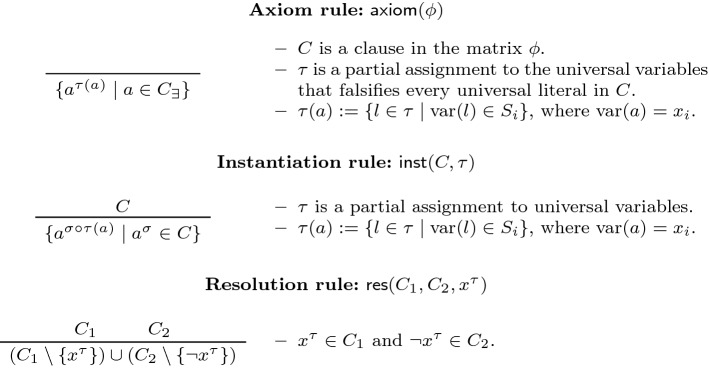
The rules of $\textsf {DQBF-IR-calc}$, where $\varPhi = {\forall u_1 \cdots \forall u_m \exists x_1(S_1) \cdots \exists x_n(S_n)}\cdot {\phi }$ is the input DQBF

Our second calculus $\textsf {DQBF-IR-calc}$ is a more sophisticated system that works with annotations representing *partial* assignments. In addition to resolution, the system is equipped with an instantiation rule with which partial annotations are ‘grown’ over the course of the derivation. For that reason, the instantiation rule uses a binary operator $\circ$ that dictates how annotations are combined, defined as follows: for any two partial assignments $\tau$ and $\sigma$ to a set of universal variables, $\tau \circ \sigma := \tau \cup \{l \in \sigma \mid \lnot l \notin \tau \}$. The rules of $\textsf {DQBF-IR-calc}$ are given in Fig. 5.

Since annotations are written as superscripts, we choose to write partial assignments not as sets, but as literal strings, e.g. $u_1\lnot u_3 \lnot u_6 u_7$. We explain the $\textsf {DQBF-IR-calc}$ rules and illustrate them with the DQBF $\varPsi$ from Example 1.

*Axiom clauses* are introduced into the proof, or *downloaded*, by selecting a clause *C* from the matrix and applying the *download assignment* to the existential literals. By design, the download assignment $\tau$ for *C* is the smallest partial assignment that falsifies every universal literal in *C*; that is, $\tau = \{\lnot l \mid l \in C_\forall \}$. When applying the download assignment, existentials are annotated only with universals in their support set. For example, downloading the the clauses $\{x_1,x_2,u_1\}$ and $\{x_1,\lnot x_2,\lnot u_1,u_2\}$ gives rise to axioms $\{x_1^{\lnot u_1}, x_2\}$ and $\{x_1^{u_1},\lnot x_2^{\lnot u_2}\}$.

*Instantiation* allows partial assignments to be combined during the course of the proof. A single partial assignment $\tau$ is applied to all the literals in the clause. As in the axiom rule, any universal variable absent from the support set is omitted from the annotation. For example, instantiating our axiom clause $\{x_1^{\lnot u_1}, x_2\}$ with $\tau = u_1 u_2$, we derive $\{x_1^{\lnot u_1}, x_2^{u_2}\}$. Note that $u_1$ does not appear in the annotation to literal $x_2$, since $u_1$ is not in the support set of variable $x_2$. Also note that literal $u_1$ does not appear in the annotation to $x_1$, which is already annotated with the negated literal $\lnot u_1$ before the instantiation takes place (see the earlier definition of the $\circ$ operator).

*Resolution* in $\textsf {IR-calc}$ is identical to propositional resolution. We emphasise that annotations are labelling distinct variables, so that a resolution step is valid only if the annotations of the pivot literals match exactly.

**Fig. 6:**
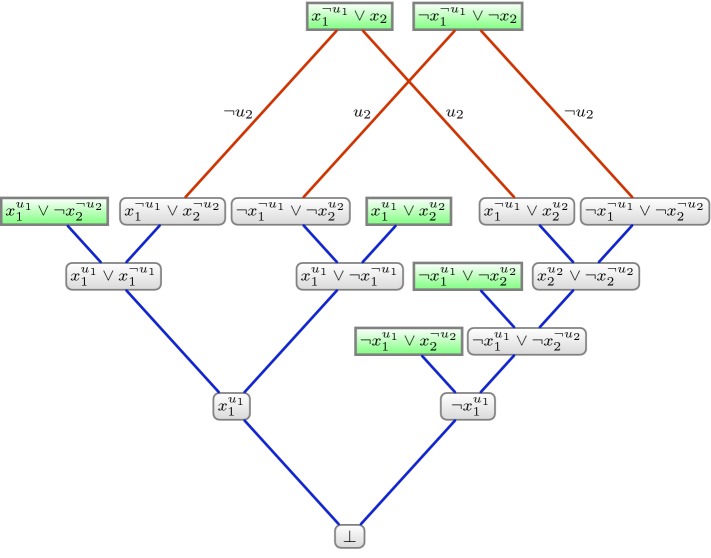
A $\textsf {DQBF-IR-calc}$ refutation of the DQBF $\varPhi$ from Example 1

A complete $\textsf {IR-calc}$ refutation of $\varPsi$ is shown in Fig. 6. We emphasise that the annotation to variable *x* never features a universal variable not in the support set of *x*. Hence, whenever $x^\tau$ is written, it is considered implicit that each variable in $\tau$ is in the support set of *x*.

*Translation to First-Order Logic* Before analysing $\textsf {DQBF-IR-calc}$ further we present the translation of DQBF into $\mathsf {EPR}$. $\mathsf {EPR}$ stands for Effectively Propositional Logic, also referred to as the Bernays–Schönfinkel fragment of first-order logic [25], characterised as the clausal language allowing only constants as function symbols (i.e. function symbols of arity larger than zero are not allowed).

We use an adaptation of the translation described for QBF [33], which becomes straightforward in the light of the DQBF semantics based on Skolem functions. The key observation is that for the intended two-valued Boolean domain the Skolem functions can in first-order logic be represented by predicates.

To translate a DQBF $\varPhi := {\mathcal {Q}}\cdot {\phi }$ with $\mathcal {Q}:= \forall u_1 \cdots \forall u_m \exists x_1(S_1) \cdots \exists x_n(S_n)$, we introduce on the first-order side (1) a predicate symbol *p* of arity one and two constant symbols 0 and 1 to describe the Boolean domain, (2) for every existential variable $x_i$ we introduce a predicate symbol $x_i$ of arity $|S_i|$, and (3) for every universal variable *u* a first-order variable *u*.

Now we can define a translation mapping $t_\mathcal {Q}$. It translates each occurrence of an existential variable $x_i$ with support set $S_i = \{u_{\rho (1)},\ldots ,u_{\rho (k)}\}$ (for some injection $\rho :[k] \rightarrow [m]$) to the atom $t_\mathcal {Q} (x) := x(u_{\rho (1)},\ldots ,u_{\rho (k)})$ (here $\rho$ represents an arbitrary but fixed order on the dependencies which dictates their placement as arguments) and each occurrence of a universal variable $u_i$ to the atom $t_\mathcal {Q} (u_i) := p(u_i)$. The mapping is then homomorphically extended to formulas: $t_\mathcal {Q} (\lnot \phi ) := \lnot t_\mathcal {Q} (\phi )$, $t_\mathcal {Q} ( \phi _1 \vee \phi _2) := t_\mathcal {Q} (\phi _1) \vee t_\mathcal {Q} (\phi _2)$, and $t_\mathcal {Q} ( \phi _1 \wedge \phi _2) := t_\mathcal {Q} (\phi _1) \wedge t_\mathcal {Q} (\phi _2)$. This means a CNF matrix $\phi$ is mapped to a corresponding first-order CNF $t_\mathcal {Q} (\phi )$. As customary, the first-order variables of $t_\mathcal {Q} (\phi )$ are assumed to be implicitly universally quantified at the top level.

#### Lemma 1

([33]) A DQBF ${\mathcal {Q}}\cdot {\phi }$ is true if and only if the first-order formula $t_\mathcal {Q} (\phi ) \wedge p(1) \wedge \lnot p(0)$ is satisfiable.

#### Proof

When the DQBF ${\forall u_1 \cdots \forall u_m \exists x_1(S_1) \cdots \exists x_n(S_n)}\cdot {\phi }$ is true, this is witnessed by the existence of Skolem functions $F=\{f_i \mid i \in [n]\}$. On the other hand, if $t_\mathcal {Q} (\phi ) \wedge p(1) \wedge \lnot p(0)$ is satisfiable then we can, by Herbrand’s theorem, assume it has a Herbrand model *H* over the base $\{0,1\}$. We can naturally translate between one and the other by setting $f_i(\mathbf {v}) = 1 \text { iff } x_i(\mathbf {v}) \in H$ for every $i \in [n]$ and $\mathbf {v} \in \{0,1\}^{|S_i|}$. The lemma then follows by structural induction over $\phi$. $\square$

For the purpose of analysing $\textsf {DQBF-IR-calc}$, the mapping $t_\mathcal {Q}$ is further extended to annotated literals: $t_\mathcal {Q} (x^\tau ) = t_\mathcal {Q} (x)\tau$ for an existential variable *x*. Here we slightly abuse notation and treat $\tau$, an annotation in the propositional context, as a first-order substitution over the corresponding translated variables in the first-order context (recall point (3) above). For example if $S_1 = \{u_1,u_2,u_3\}$ then $t_\mathcal {Q} (x_1^{\lnot u_1, u_2 }) = x_1(u_1,u_2,u_3)\{u_1 \mapsto 0, u_2 \mapsto 1\} = x_1(0,1,u_3)$.

*Soundness and Completeness* We aim to show soundness and completeness of $\textsf {DQBF-IR-calc}$ by relating it via the above translation to a first-order resolution calculus FO-Res. This calculus consists of (1) an instantiation rule: given a clause *C* and a substitution $\sigma$ derive the instance $C\sigma$, and (2) the resolution rule: given two clauses $C \cup \{l\}$ and $D \cup \{\lnot l\}$, where *l* is a first-order literal, derive $C \cup D$. Note that similarly to propositional clauses, we understand first-order clauses as *sets* of literals. Thus we do not need any explicit factoring rule. Also note that we require the resolved pivots of the two premises of the resolution rule to be equal (up to the polarity). Standard first-order resolution, which involves *unification* of the resolved literals, can be simulated in FO-Res by combining the instantiation and the resolution rule.

It is well known that FO-Res is sound and complete for first-order logic. For the proof of Theorem 5 below, we will work with a more specific calculus known as *ordered resolution* [2]. Ordered resolution FO-Res$^<$ is parametrised by an ordering < on symbols, which is extended to literals in a natural way and only maximal literals in each clause are eligible as pivots. Also ordered resolution is complete [2].

#### Theorem 4

$$\textsf {DQBF-IR-calc}$$ is sound.

#### Proof

Given $\pi = C_1, \dots , C_n$, a $\textsf {DQBF-IR-calc}$ derivation of the empty clause $C_n = \bot$ from the DQBF ${\mathcal {Q}}\cdot {\phi }$, we show by induction that $t_\mathcal {Q} (C_i)$ is derivable from $\varPhi = t_\mathcal {Q} (\phi ) \wedge p(1) \wedge \lnot p(0)$ by FO-Res for every $i\le n$. Because $t_\mathcal {Q} (\bot )=\bot$ is unsatisfiable, so must be $\varPhi$, by the soundness of FO-Res, and therefore $\mathcal {Q} \cdot \phi$ is false by Lemma 1. We consider the three cases by which a clause $C_i$, $i \le n$, can be derived by $\textsf {DQBF-IR-calc}$.

First, let us assume that $C_i$ follows by instantiation, i.e., $C_i = {\textsf {inst}}(C_j,\tau )$ for some $j < i$ and an annotation $\tau$. By the inductive hypothesis, we know that $t_\mathcal {Q} (C_j)$ is derivable from $\varPhi$ by FO-Res. The case follows by observing that $t_\mathcal {Q} ({\textsf {inst}}(C_j,\tau )) = t_\mathcal {Q} (C_j)\tau$ and thus $t_\mathcal {Q} (C_i)$ can be derived from $t_\mathcal {Q} (C_i)$ by first-order instantiation with $\tau$ understood as a substitution.

Second, it is easy to see that if $C_i$ follows from $C_j$ and $C_k$ by the $\textsf {DQBF-IR-calc}$ resolution rule, $t_\mathcal {Q} (C_i)$ can be derived from $t_\mathcal {Q} (C_j)$ and $t_\mathcal {Q} (C_k)$ by FO-Res.

Third, let us assume that $C_i$ is derived using the Axiom rule of $\textsf {DQBF-IR-calc}$. This means that

$$\begin{aligned} C_i = \{a^{\tau (a)} \mid a \in C_\exists \}, \end{aligned}$$

where $C \in \phi$ and $\tau (a) = \{\lnot l \mid l \in C_\forall , \text{ var }(l) \in S_i \}$ for $\text{ var }(a) = x_i$. To derive $t_\mathcal {Q} (C_i)$ from $\varPhi$ by FO-Res, we first instantiate $t_\mathcal {Q} (C)$ by $\tau = \{\lnot l \mid l \in C_\forall \}$ obtaining $D = t_\mathcal {Q} (C)\tau$. Next we observe that for any $a \in C_\exists$ we have $t_\mathcal {Q} (a)\tau = t_\mathcal {Q} (a^{\tau (a)})$ and for any $l \in C_\forall$ we have $t_\mathcal {Q} (l)\tau \in \{p(0), \lnot p(1)\}$ by the definition of $\tau$. Thus $t_\mathcal {Q} (C_i) \subseteq D \subseteq t_\mathcal {Q} (C_i) \cup \{p(0), \lnot p(1)\}$. We derive $t_\mathcal {Q} (C_i)$ from *D* in FO-Res by resolving *D* with the available units *p*(1) and $\lnot p(0)$ if needed. $\square$

#### Example 3

To demonstrate the third case of the above proof, let us take a DQBF with a prefix $\mathcal {Q} = \forall u \forall v \forall w \exists x(u,v) \exists y(v,w)$. Below, the Axiom rule is applied to a clause $C = \{x, y ,\lnot u, v \}$ on the left and a corresponding first-order step to its translation $t_\mathcal {Q} (C)$ on the right. 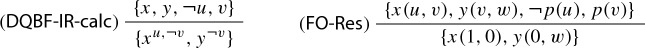


#### Theorem 5

$$\textsf {DQBF-IR-calc}$$ is complete.

#### Proof

Let ${\mathcal {Q}}\cdot {\phi }$ be a false DQBF and let us consider $\mathcal {G}(t_\mathcal {Q} (\phi ))$, the set of all ground instances of clauses in $t_\mathcal {Q} (\phi )$. Here, by a ground instance of a clause *C* we mean the clause $C\tau$ for some substitution $\tau :\text{ var }(C)\rightarrow \{0,1\}$. By the combination of Lemma 1 and Herbrand’s theorem, $\mathcal {G}(t_\mathcal {Q} (\phi )) \wedge p(1) \wedge \lnot p(0)$ is unsatisfiable and thus it has a FO-Res$^<$ refutation for any eligible symbol ordering <. We can chose the ordering such that literals containing predicate *p* become maximal in their respective clauses. This choice then has the following consequences for the refutation: (1) the refutation does not contain clauses subsumed by *p*(1) or $\lnot p(0)$, and (2) any clause containing the predicate *p* is resolved on a literal containing *p*. From this it is easy to see that any leaf in the refutation gives rise (in zero, one or two resolution steps with *p*(1) or $\lnot p(0)$) to a clause $D = t_\mathcal {Q} (C)$ where *C* can be obtained by $\textsf {DQBF-IR-calc}$ axiom from a $C^\prime \in \phi$, possibly followed by instantiation. The rest of the refutation consists of FO-Res$^<$ resolution steps which can be simulated by $\textsf {DQBF-IR-calc}$ in a one-to-one fashion (unlike FO-Res$^<$, $\textsf {DQBF-IR-calc}$ does not have any ordering restrictions). $\square$

Although one can lift the above argument with ordered resolution to show that the set

$$\begin{aligned} \{t_\mathcal {Q} (C) \mid C \text{ can } \text{ be } \text{ derived } \text{ by } {\textsf {axiom}}(\phi )\} \end{aligned}$$

is unsatisfiable for any false DQBF ${\mathcal {Q}}\cdot {\phi }$, we have only shown how to simulate *ground*FO-Res$^<$ steps by $\textsf {DQBF-IR-calc}$. That is because a lifted FO-Res$^<$ derivation may contain instantiation steps which *rename variables apart* for which a subsequent resolvent cannot be represented in $\textsf {DQBF-IR-calc}$. An example is the resolvent $\{{y}({v}) ,{z}({v'})\}$ of clauses $\{x(u), y(v)\}$ and $\{\lnot {x}({u}) , {z}({v}')\}$ which is obviously stronger than the clause $\{{y}({v}) , {z}({v})\}$. However, only the latter has a counterpart in $\textsf {DQBF-IR-calc}$.

We also remark that in a similar way we can also lift to DQBF the QBF calculus $$\forall $$$$\textsf {Exp+Res}$$ from [23]. It is easily verified that the simulation of $$\forall $$$$\textsf {Exp+Res}$$ by $$\textsf {IR-calc}$$ shown in [7] directly transfers from QBF to DQBF. Hence Theorem 5 immediately implies the soundness of $$\forall $$$$\textsf {Exp+Res}$$ lifted to DQBF. Moreover, because all ground instances are also available in $$\forall $$$$\textsf {Exp+Res}$$ lifted to DQBF, this system is also complete as can be shown by repeating the argument of Theorem 5.

#### Corollary 1

$$\textsf {DQBF-}$$$$\forall $$$$\textsf {Exp+Res}$$ is sound and complete.

### Dependency Schemes in Expansion-Based QBF Systems

As we noted in Sect. 4.2, our DQBF interpretation allows a QBF calculus, when augmented with a dependency scheme, to be equated with a fragment of a suitable DQBF system using the notion of projection (Definition 5). This idea remains appropriate in the current context, in which we propose $\textsf {IR}(\mathcal {D})\textsf {-calc}$, the natural parameterisation of $\textsf {IR-calc}$ by a dependency scheme, whereupon Theorem 4 has direct consequences for soundness. As it turns out, in contrast to $\textsf {QU}(\mathcal {D})\textsf {-Res}$, full exhibition characterises precisely the schemes for which $\textsf {IR}(\mathcal {D})\textsf {-calc}$ is sound.

Similarly as for $\textsf {QU-Res}$, the natural generalisation of $\textsf {IR-calc}$ by dependency scheme has exactly the same logical rules as the (hypothetical) system in which one first maps the instance to its image under $\mathcal {D}$, and then uses the rules of $\textsf {DQBF-IR-calc}$. For that reason, the $\textsf {IR}(\mathcal {D})\textsf {-calc}$ derivations from $\varPhi$ are exactly the $\textsf {DQBF-IR-calc}$ derivations from $\mathcal {D} (\varPhi )$. Thus, we define $\textsf {IR}(\mathcal {D})\textsf {-calc}$ as the appropriate projection of $\textsf {DQBF-IR-calc}$.

#### Definition 7

For each dependency scheme $\mathcal {D}$, the QBF calculus $\textsf {IR}(\mathcal {D})\textsf {-calc}$ is the projection of $\textsf {DQBF-IR-calc}$ according to $\mathcal {D}$.

Our DQBF interpretation now becomes rather fruitful, interfacing directly with the connections to first-order logic exploited in the previous subsection. The following characterisation of soundness in $\textsf {IR}(\mathcal {D})\textsf {-calc}$ is almost immediate from the soundness and incompleteness of $\textsf {DQBF-IR-calc}$.

#### Theorem 6

$\textsf {IR}(\mathcal {D})\textsf {-calc}$ is sound if and only if $\mathcal {D}$ is fully exhibited.

#### Proof

If $\mathcal {D}$ is fully exhibited, it maps every true QBF $\varPhi$ to a true DQBF $\mathcal {D} (\varPhi )$. By Theorem 4, $\textsf {DQBF-IR-calc}$ is sound, and cannot refute $\mathcal {D} (\varPhi )$. Hence $\textsf {IR}(\mathcal {D})\textsf {-calc}$ cannot refute $\varPhi$.

On the other hand, if $\mathcal {D}$ is not fully exhibited, it maps some true QBF $\varPhi ^\prime$ to a false DQBF $\mathcal {D} (\varPhi ^\prime )$. By Theorem 5, $\textsf {DQBF-IR-calc}$ is complete, and can therefore refute $\mathcal {D} (\varPhi ^\prime )$. It follows that $\textsf {IR}(\mathcal {D})\textsf {-calc}$, which can refute the true QBF $\varPhi ^\prime$, is not sound. $\square$

Naturally, the situation for the base expansion system $$\forall $$$$\textsf {Exp+Res}$$ is very similar to $$\textsf {IR-calc}$$. Repeating our method, $$\forall $$$$\textsf {Exp}(\mathcal {D})\textsf {+Res}$$ can be defined as a projection of $$\textsf {DQBF-}$$$$\forall $$$$\textsf {Exp+Res}$$, and the characterisation of soundness in terms of full exhibition can be proved similarly to Theorem 6.

#### Definition 8

For each dependency scheme $$\mathcal {D}$$, the QBF calculus $$\forall $$$$\textsf {Exp}(\mathcal {D})\textsf {+Res}$$ is the projection of $$\textsf {DQBF-IR-calc}$$ according to $$\mathcal {D}$$.

#### Theorem 7

$$\forall $$$$\textsf {Exp}(\mathcal {D})\textsf {+Res}$$ is sound if and only if $$\mathcal {D}$$ is fully exhibited.

## Demonstrating Full Exhibition

In this section, we demonstrate that the reflexive resolution path dependency scheme $\mathcal {D}^{\textsf {rrs}}$ [37] is fully exhibited, thereby proving the conjecture of Slivovsky [35, p. 37]. We begin by recalling $\mathcal {D}^{\textsf {rrs}}$ in Sect. 6.1, and proceed to prove the result in Sect. 6.2.

### Definition of the Dependency Scheme $\mathcal {D}^{\textsf {rrs}}$

The reflexive resolution path dependency scheme $\mathcal {D}^{\textsf {rrs}}$ is arguably the most important in the literature, being the most general scheme $\mathcal {D}$ for which it is known that $\textsf {Q}(\mathcal {D})\textsf {-Res}$ is sound. Given that we consider only the (in)dependence of existentials on universals (see the discussion in Sect. 3.1), we obtain a presentation of $\mathcal {D}^{\textsf {rrs}}$ that is significantly simpler than the original definition in [37].

The scheme works by appeal to the syntactic form of an instance, and identifies dependent variables by means of connections via matrix clauses. These connections, known as *resolution paths* [40], associate pairs of clauses in which some connecting existential variable appears in opposite polarities. For our purposes in the present work, we capture a refined notion of resolution path with the binary relation $\mathcal {C}$.

#### Definition 9

(Adapted from [40]) Let $\varPhi ={\forall u_1 \cdots \forall u_m \exists x_1(S_1) \cdots \exists x_n(S_n)}\cdot {\phi }$ be a QBF, let $i \in [m]$ and $j \in [i]$, and let *l* and $l^\prime$ be literals with $\text{ var }(l) = u_i$ and $\text{ var }(l^\prime ) = x_j$. A sequence of clauses $C_1, \dots ,C_p \in \phi$ with $l \in C_1$ and $l^\prime \in C_p$ is a *resolution path in*$\varPhi$*fromlto*$l^\prime$ iff (a) $u_i \in S_j$, and (b) there is a sequence of existential literals $l_1, \dots , l_{p-1}$ for which the following three conditions hold:

(i) $u_i$ is in the support set of $\text{ var }(l_k)$, for each $k \in [p-1]$,
(ii) $l_k \in C_k$ and $\lnot l_k \in C_{k+1}$, for each $k \in [p-1]$,
(iii) $\text{ var }(l_k) \ne \text{ var }(l_{k+1})$ for each $k \in [p-2]$.

We say that $(l,l^\prime ) \in \mathcal {C}_\varPhi$ if and only if there exists a resolution path in $\varPhi$ from *l* to $l^\prime$.

We emphasise that, in a resolution path from a (universal) literal *l* to an (existential) literal $l^\prime$ (with $\text{ var }(l)$ in the support set of $\text{ var }(l^\prime )$), $\text{ var }(l)$ should appear in the support set of each ‘connecting’ existential variable $\text{ var }(l_1), \dots , \text{ var }(l_{p-1})$. Now, given an existential *x* and a universal *u* in the support set of *x*, $\mathcal {D}^{\textsf {rrs}}$ determines that *x* depends on *u* by finding a suitable pair of resolution paths; namely, one resolution path from a literal in *u* to a literal in *x*, and a second path between the complementary literals.

#### Definition 10

($\mathcal {D}^{\textsf {rrs}}$ [37]) Let $\varPhi ={\forall u_1 \cdots \forall u_m \exists x_1(S_1) \cdots \exists x_n(S_n)}\cdot {\phi }$ be a QBF. The *reflexive resolution path dependency scheme*$\mathcal {D}^{\textsf {rrs}}$ is defined by

$$\begin{aligned} \mathcal {D}^{\textsf {rrs}} (\varPhi ) = {\forall u_1 \cdots \forall u_m \exists x_1(S^\prime _1) \cdots \exists x_n(S^\prime _n)}\cdot {\phi } \end{aligned}$$

in which $u_i \in S^\prime _j$ if and only if (a) $u_i \in S_j$, and (b) there exist literals *l* and $l^\prime$ with $\text{ var }(l) = u_i$ and $\text{ var }(l^\prime ) = x_j$ for which $(l,l^\prime ) \in \mathcal {C}_\varPhi$ and $(\lnot l, \lnot l^\prime ) \in \mathcal {C}_\varPhi$.

### Proof of the Full Exhibition of $\mathcal {D}^{\textsf {rrs}}$

In order to prove that $\mathcal {D}^{\textsf {rrs}}$ is fully exhibited, we must show that the image under $\mathcal {D}^{\textsf {rrs}}$ of an arbitrary true QBF $\varPhi$ is a true DQBF $\mathcal {D}^{\textsf {rrs}} (\varPhi )$. Our method involves taking an arbitrary model for $\varPhi$ and transforming it, step by step, into a model for $\mathcal {D}^{\textsf {rrs}} (\varPhi )$.

To do so, we need a framework and notation for manipulating DQBF models. We choose to work with assignment trees [32]. An assignment tree can be naturally interpreted as a set of *paths* (not to be confused with resolution paths), where each path from the root of the tree to a leaf spells out a total assignment to the variables of the formula. In this context, a *path* for a DQBF $\varPhi$ is a set containing exactly one literal for each variable in $\varPhi$ (and nothing more). We will be frequently inspecting the literals appearing in paths, so we adopt the following notation: For a variable *z* and path *P*, we write *P*[*z*] for the unique literal *l* in *P* with $\text{ var }(l) = z$; that is, if $\lnot z \in P$, then $P[z] = \lnot z$, otherwise $P[z] = z$.

In order for a set of paths to properly represent an assignment tree for a DQBF, it must contain exactly one path for each total assignment to the universal variables, and must also respect the variable dependencies given by the prefix support sets. Additionally, for an assignment tree to represent a model, every path must satisfy every clause of the matrix. We give a formal definition below.

#### Definition 11

(Assignment tree) Let $\varPhi = {\forall u_1 \cdots \forall u_m \exists x_1(S_1) \cdots \exists x_n(S_n)}\cdot {\phi }$ be a DQBF. A set *T* of paths for $\varPhi$ is an *assignment tree for*$\varPhi$ if and only if the following two conditions hold:

- For each total assignment *U* to $\{u_1, \dots ,u_m\}$, there is a unique path in *T* that contains *U*.
- For each $i \in [m]$, for each $j \in [n]$ with $u_i \notin S_j$, and for each path $P \in T$, $P[x_j] = \text{ comp }(P,T,u_i)[x_j]$,

where the path complementary to *P* in *T* with respect to $u_i$, written $\text{ comp }(P,T,u_i)$, is the unique path in *T* that agrees with *P* on all universal variables except $u_i$. Additionally, *T* represents a model for $\varPhi$ if and only if every path in *T* satisfies every clause in $\phi$.

The second item in the above definition is of particular prominence in the following proofs. Therefore, for the sake of readability, if condition (b) holds for an assignment tree *T*, we say that ‘*T* exhibits the independence of $x_j$ on $u_i$’. We will often write ‘*T* models $\varPhi$’ rather than ‘*T* represents a model for $\varPhi$’.

We may now reformulate DQBF semantics in terms of assignment trees. Where an assignment tree represents a model we will prefer to use the symbol *M* as opposed to *T*.

#### Proposition 4

A DQBF $\varPhi$ is true if and only if there is an assignment tree *M* that models $\varPhi$.

Our proof method may be described as follows. We take an arbitrary model *M* for $\varPhi$. Given that $\mathcal {D}^{\textsf {rrs}} (\varPhi )$ is stronger than $\varPhi$, there are extra independencies that need to be exhibited if *M* is to be transformed into a model for $\mathcal {D}^{\textsf {rrs}} (\varPhi )$. We achieve this by working independently with each universal variable in turn. That is, we first transform *M* into a model $M_1$ that exhibits all the required existential independencies on $u_1$, then into a model $M_2$ that exhibits all those on $u_2$, and so on through to the final universal $u_m$, whereupon $M_m$ models $\mathcal {D}^{\textsf {rrs}} (\varPhi )$.

We transform models by working with the individual paths, for which we introduce the following notion of *reformed path*. A path *P* is reformed with respect to universal variable *u* by copying particular existential assignments from its corresponding complementary path. Specifically, for any existential *x* whose support set contains variable *u*, a path $P \in M$ takes the *x*-literal from the complementary path $\text{ comp }(P,M,u)$ if and only if there is no resolution path from the literal $\lnot P[u]$ to the negation of that *x*-literal. Reforming a path does not affect the universal literals.

#### Definition 12

(Reformed path) Let *M* be a model for a DQBF $\varPhi$, let *P* be a path in *M*, let *u* be a universal variable in $\varPhi$ and put $l = P[u]$. The *reformed path*$\text{ ref }(P,M,u)$*ofPinMwith respect tou* is given by

$$\begin{aligned} \text{ ref }(P,M,u)[z] = {\left\{ \begin{array}{ll} \text{ comp }(P,M,u)[z] &{}\quad \text{ if } z \text{ is } \text{ existential } \text{ and } (\lnot l,\lnot l^\prime ) \notin \mathcal {C}_\varPhi \,,\\ P[z] &{}\quad \text{ otherwise } \,, \end{array}\right. } \end{aligned}$$

where *z* is an arbitrary variable appearing in $\varPhi$ and $l^\prime = \text{ comp }(P,M,u)[z]$.

In what follows, it is crucial that reforming any path in a model for a DQBF does not cause that path to falsify any clauses in the matrix. That is the subject of the following lemma.

#### Lemma 2

Let *M* be a model for a DQBF $\varPhi$, let *P* be a path in *M* and let *u* be a universal variable appearing in $\varPhi$. Then $\text{ ref }(P,M,u)$ satisfies every clause in the matrix of $\varPhi$.

#### Proof

Let $\varPhi = {\mathcal {Q}}\cdot {\phi }$. Aiming for contradiction, we suppose that $\text{ ref }(P,M,u)$ falsifies some clause $C \in \phi$. We show that $\text{ comp }(P,M,u)$ falsifies *C*, contradicting the fact that *M* is a model.

Noting that *P* and $\text{ ref }(P,M,u)$ agree on universal variables, we assume w.l.o.g. that $P[u] = \text{ ref }(P,M,u)[u] = \lnot u$ and $\text{ comp }(P,M,u)[u] = u$. Also, since *P* satisfies *C*, there exists an existential variable *x* appearing in *C* on which *P* and $\text{ ref }(P,M,u)$ disagree. Hence, assuming w.l.o.g. that *x* appears positively in *C*, we have $P[x] = x$ and $\text{ ref }(P,M,u)[x] = \lnot x$. It follows then, from the definition of reformed path, that $\text{ comp }(P,M,u)[x] = \lnot x$ and that

1 $$\begin{aligned} (u,x) \notin \mathcal {C}_\varPhi . \end{aligned}$$

Now we show that $\text{ comp }(P,M,u)$ falsifies *C*, or, equivalently, that the intersection $\text{ comp }(P,M,u) \cap C$ is empty.

We first show that $\text{ comp }(P,M,u)$ contains none of the universal literals in *C*. Note that, since $x \in C$, we cannot have $u \in C$, for that would imply $(u,x) \in \mathcal {C}_\varPhi$ by definition of $\mathcal {D}^{\textsf {rrs}}$, contradicting statement (1). Also, $\text{ ref }(P,M,u)$ does not contain any of the universal literals in *C*, and $\text{ comp }(P,M,u)$ agrees with $\text{ ref }(P,M,u)$ on all universal variables other than *u*. Hence $\text{ comp }(P,M,u) \cap C_\forall = \emptyset$.

Finally, we show that $\text{ comp }(P,M,u)$ contains none of the existential literals in *C*. Let *l* be any existential literal in *C* with $\text{ var }(l) \ne x$. If $(u, \lnot l) \in \mathcal {C}_\varPhi$, then there exists a sequence of clauses $C_1, \dots ,C_k \in \phi$ and a sequence of existential literals $l_1, \dots ,l_{k-1}$ satisfying the four conditions of Definition 9. Then, it is readily verifiable that the sequences $C_1, \dots ,C_k,C \in \phi$ and $l_1, \dots ,l_{k-1},l$ also satisfy Definition 9, showing that $(u,x) \in \mathcal {C}_\varPhi$ and contradicting statement (1). It follows that $(u, \lnot l) \notin \mathcal {C}_\varPhi$, and that

2 $$\begin{aligned} \text{ comp }(P,M,u)[\text{ var }(l)] = l \qquad \text {implies} \qquad \text{ ref }(P,M,u)[\text{ var }(l)] = l \,, \end{aligned}$$

by the definition of reformed path. However, $\text{ ref }(P,M,u)[\text{ var }(l)] = \lnot l$, since the reformed path falsifies *C* by supposition, and hence $\text{ comp }(P,M,u)[\text{ var }(l)] = \lnot l$, by the contrapositive of (2). Since we noted earlier that $\text{ comp }(P,M,u)[x] = \lnot x$, it follows that $\text{ comp }(P,M,u) \cap C_\exists = \emptyset$. $\square$

The main idea behind the model transformation is that every path $P \in M$ will be reformed with respect to *u*, essentially by comparison with its complementary path $\text{ comp }(P,M,u)$, and that the result will exhibit the necessary existential independencies on *u*. It is quite natural to perform this procedure in two stages. First, all paths containing literal $\lnot u$ are reformed, and we call the result the *left reform*^5^ of *M* with respect *u*. We then reform the remaining paths, i.e. we take the *right reform* of the resulting model. The two-stage process reflects the way that $\mathcal {D}^{\textsf {rrs}}$ works with polarity, and requires the existence of a *pair* of resolution paths to determine the dependency relation.

#### Definition 13

(Reformed model) Let *M* be a model for a DQBF $\varPhi$, let *u* be a universal variable in $\varPhi$, and let $L = \{P \in M \mid P[u] = \lnot u\}$ (respectively $R = \{P \in M \mid P[u] = u\}$). The *left-reform* (respectively *right-reform*) *of M with respect to u* is given by the set of paths $(M {\setminus } L) \cup \{\text{ ref }(P,M,u) \mid P \in L\}$ (respectively $(M {\setminus } R) \cup \{\text{ ref }(P,M,u) \mid P \in R\}$). The *reformed model*$\text{ ref }(M,u)$ of *M* with respect to *u* is the right reform of $M^\prime$ with respect to *u*, where $M^\prime$ is the left reform of *M* with respect to *u*.

From the definition of a reformed model, it follows that left-reform of a model with respect to *u* preserves all paths that contain the positive literal *u*; similarly, right-reform preserves all paths containing the negative literal $\lnot u$. We use this property fairly frequently in the forthcoming proofs of lemmas 4 and 5.

In order to prove our main result, we need to prove three properties of the reformed model. First, it is of course necessary that the reformed model is indeed a model, as stated in the following lemma.

#### Lemma 3

Let $\varPhi$ be a QBF, and let *u* be a universal variable appearing in $\varPhi$. If *M* models $\varPhi$, then $\text{ ref }(M,u)$ also models $\varPhi$.

#### Proof

Let $\varPhi = {\forall u_1 \cdots \forall u_m \exists x_1 (S_1) \cdots \exists x_n (S_n)}\cdot {\phi }$, and let $M^\prime$ be the left-reform of *M* with respect to *u*. We show that $M^\prime$ is an assignment tree for $\varPhi$. It is clear that $M^\prime$ is a set of paths for $\varPhi$, so we verify that $M^\prime$ satisfies conditions (a) and (b) of Definition 11.

Since *M* is an assignment tree for $\varPhi$, for each total assignment *U* to the universal variables $\{u_1, \dots ,u_m\}$, there is a unique path in *M* that contains *U*, by condition (a). By definition, reforming a path does not affect the universal literals, so $M^\prime$ also satisfies condition (a).

To see that $M^\prime$ satisfies condition (b), let $i \in [m]$ and $j \in [n]$ such that $u_i \notin S_j$. Further, let $P^\prime$ and $Q^\prime$ be paths in $M^\prime$ such that $Q^\prime = \text{ comp }(P^\prime ,M^\prime ,u_i)$, and assume w.l.o.g. that $P^\prime [u_i] = \lnot u_i$ and $Q^\prime [u_i] = u_i$. We only need to show that $P^\prime [x_j] = Q^\prime [x_j]$. To that end, let *P* and *Q* be the unique paths in *M* that agree with $P^\prime$ and $Q^\prime$ respectively on all universal variables. We observe that $Q = \text{ comp }(P,M,u_i)$, and since *M* models $\varPhi$ we have $P[x_j] = Q[x_j]$. We consider two cases.

1. Suppose that $u = u_i$. Then $P^\prime = \text{ ref }(P,M,u_i)$ (since $P[u_i] = \lnot u_i$) and $Q^\prime = Q$ (since $Q[u_i]=u_i$). By definition of reformed path, $P^\prime [x_j]$ is equal to either $P[x_j]$ or $Q[x_j]$, and in either case $P^\prime [x_j] = Q^\prime [x_j]$.
2. Suppose instead that $u \ne u_i$. We consider two subcases.
  - Suppose that $P[u] = Q[u] = \lnot u$. By definition of left reform, we must have $P^\prime = \text{ ref }(P,M,u)$ and $Q^\prime = \text{ ref }(Q,M,u)$, so it suffices to show that $\text{ ref }(P,M,u)[x_j] = \text{ ref }(Q,M,u)[x_j]$. Note that the paths $\text{ comp }(P,M,u)$ and $\text{ comp }(Q,M,u)$ are complementary in *M* with respect to $u_i$, and since *M* models $\varPhi$ and $u_i \notin S_j$ we have $\text{ comp }(P,M,u)[x_j] = \text{ comp }(Q,M,u)[x_j]$. Now, if $(u,x_j) \notin C_\varPhi$, then we have $\text{ ref }(P,M,u)[x_j] = \text{ comp }(P,M,u)[x_j]$ and $\text{ ref }(Q,M,u)[x_j] = \text{ comp }(Q,M,u)[x_j]$. Otherwise, if $(u,x_j) \in C_\varPhi$, then $\text{ ref }(P,M,u)[x_j] = P[x_j]$ and $\text{ ref }(Q,M,u)[x_j] = Q[x_j]$. In either case, we have $\text{ ref }(P,M,u)[x_j] = \text{ ref }(Q,M,u)[x_j]$.
  - Suppose on the other hand that $P[u] = Q[u] = u$. Then $P^\prime = P$ and $Q^\prime = Q$ by definition of left reform, and it follows immediately that $P^\prime [x_j] = Q^\prime [x_j]$.

By Lemma 2, every path in $M^\prime$ satisfies every clause in $\phi$, therefore $M^\prime$ models $\varPhi$. A similar argument shows that the reformed model of *M* with respect to *u*, which is the right-reform of $M^\prime$ with respect to *u*, also models $\varPhi$. $\square$

Secondly, we need to prove that the reformed model (with respect to *u*) does in fact exhibit the existential independencies on *u* identified by $\mathcal {D}^{\textsf {rrs}}$. That is, we must show that the reformed model does indeed exhibit the independence of *x* on *u* whenever *u* is absent from the support set of *x* in the DQBF $\mathcal {D}^{\textsf {rrs}} (\varPhi )$. We state this formally in the following lemma. The fact that this lemma holds is exactly the motivation behind the construction of the reformed path.

#### Lemma 4

Let *M* be a model for a QBF $\varPhi = {\forall u_1 \cdots \forall u_m \exists x_1 (S_1) \cdots \exists x_n (S_n)}\cdot {\phi }$, let $\mathcal {D}^{\textsf {rrs}} (\varPhi ) = {\forall u_1 \cdots \forall u_m \exists x_1 (S^\prime _1) \cdots \exists x_n (S^\prime _n)}\cdot {\phi }$, and let $i \in [m]$. For each $j \in [n]$ with $u_i \notin S^\prime _j$, $\text{ ref }(M,u_i)$ exhibits the independence of $x_j$ on $u_i$.

#### Proof

Let $M^\prime$ be the left reform of *M* with respect to $u_i$, and let $M^{{\prime \prime }} = \text{ ref }(M,u_i)$ be the right reform of $M^\prime$ with respect to $u_i$. Further, let *U* be an arbitrary (total) assignment to the universals $\{u_1, \dots ,u_m\}$, and denote by *P*, $P^\prime$ and $P^{\prime \prime }$ the unique paths in *M*, $M^\prime$ and $M^{\prime \prime }$ (respectively) that contain *U*. Also, let *Q*, $Q^\prime$ and $Q^{\prime \prime }$ be the paths complementary to *P*, $P^\prime$ and $P^{\prime \prime }$ in *M*, $M^\prime$ and $M^{\prime \prime }$ (respectively) with respect to $u_i$.

Now let $j \in [n]$ such that $u_i \notin S^\prime _j$. Since *U* is an arbitrary total assignment to the universals that defines a unique path in a model for $\varPhi$, showing that $\text{ ref }(M,u_i)$ exhibits the independence of $x_j$ on $u_i$ is equivalent to showing $P^{\prime \prime }[x_j] = Q^{\prime \prime }[x_j]$. Hence we may assume w.l.o.g. that $\lnot u_i \in U$, so $P[u_i] = P^\prime [u_i] = P^{\prime \prime }[u_i] = \lnot u_i$ and $Q[u_i] = Q^\prime [u_i] = Q^{\prime \prime }[u_i] = u_i$. We consider two cases.

1. Suppose that $P[x_j] = Q[x_j]$. Since $M^\prime$ is the left reform of *M* with respect to $u_i$, and left reform with respect to $u_i$ preserves any path containing $u_i$, we have $Q^\prime = Q$. Since *P* and *Q* agree on $x_j$, the reformed path $\text{ ref }(P,M,u_i)$ must also agree with both *P* and *Q* on $x_j$, by definition of reformed path. Hence $P^\prime [x_j] = Q^\prime [x_j]$, which implies $P^{\prime \prime }[x_j] = Q^{\prime \prime }[x_j]$ unconditionally (i.e., it does not rely on $P[x_j] = Q[x_j]$). To see this, note that $Q^{\prime \prime }= \text{ ref }(Q^\prime ,M^\prime ,u_i)$, and hence $Q^{\prime \prime }[x_j] = P^\prime [x_j]$ by definition of reformed path. Moreover, $P^{\prime \prime }= P^\prime$ because $P^\prime [u_i] = \lnot u_i$, and right reform with respect to $u_i$ preserves any path containing $\lnot u_i$.
2. On the other hand, suppose that $P[x_j] \ne Q[x_j]$. Further, assume w.l.o.g. that $P[x_j] = x_j$ and $Q[x_j] = \lnot x_j$. Now, since $u_i \notin S^\prime _j$, we have either $(u_i,x_j) \notin \mathcal {C}_\varPhi$ or $(\lnot u_i,\lnot x_j) \notin \mathcal {C}_\varPhi$, by definition of $\mathcal {D}^{\textsf {rrs}}$, so we consider two sub-cases.
  - Suppose that $(u_i,x_j) \notin \mathcal {C}_\varPhi$. Since $M^\prime$ is the left reform of *M* with respect to $u_i$, we have $P^\prime = \text{ ref }(P,M,u_i)$ (because $P[u_i] = \lnot u_i$) and $Q^\prime = Q$ (because $Q[u_i] = u_i$). Therefore $P^\prime [x_j] = Q[x_j] = Q^\prime [x_j]$ by definition of reformed path. The fact that $P^{\prime \prime }[x_j] = Q^{\prime \prime }[x_j]$ may then be deduced exactly as in case (1) above.
  - Suppose on the other hand that $(u_i,x_j) \in \mathcal {C}_\varPhi$. Then $P^\prime [x_j] = P[x_j]$ by definition of reformed path. Since $M^{\prime \prime }$ is the right reform of $M^\prime$ with respect to $u_i$, we have $P^{\prime \prime }= P^\prime$ (because $P^\prime [u_i] = \lnot u_i$) and $Q^{\prime \prime }= \text{ ref }(Q^\prime ,M^\prime ,u_i)$ (because $Q^\prime [u_i] = u_i$). Moreover, we must have $(\lnot u_i, \lnot x_j) \notin \mathcal {C}_\varPhi$, for otherwise $u_i \in S^\prime _j$ by definition of $\mathcal {D}^{\textsf {rrs}}$. Since $P^\prime [x_j] = x_j$, we have $Q^{\prime \prime }[x_j] = P^\prime [x_j]$ by definition of reformed path. Therefore $P^{\prime \prime }[x_j] = P^\prime [x_j] = Q^{\prime \prime }[x_j]$.$\square$

Now, we intend to successively reform the model *M* to exhibit independences on the universal variables, one by one. It is therefore crucial that future reforms do not undo the work of previous ones; that is, reforming a model must preserve the existing exhibition of independencies. This is indeed the case, as stated in our third and final lemma.

#### Lemma 5

Let *M* be a model for a QBF $\varPhi$. Further, let *u* and *v* be universal variables and let *x* be an existential variable, all of which appear in $\varPhi$. If *M* exhibits the independence of *x* on *v*, then so does $\text{ ref }(M,u)$.

#### Proof

Let $M^\prime$ be the left reform of *M* with respect to *u*, and let $M^{\prime \prime }= \text{ ref }(M,u)$ be the right reform of $M^\prime$ with respect to *u*. Let *U* be an arbitrary (total) assignment to the universal variables of $\varPhi$, and assume w.l.o.g. that $\{\lnot u, \lnot v \}\subseteq U$.

We define twelve paths that we work with in this proof. Let $P_{\lnot v}$, $P^\prime _{\lnot v}$ and $P^{\prime \prime }_{\lnot v}$ be the unique paths in *M*, $M^\prime$ and $M^{\prime \prime }$ (respectively) that contain *U*, and let $Q_{\lnot v}$, $Q^\prime _{\lnot v}$ and $Q^{\prime \prime }_{\lnot v}$ be paths complementary to $P_{\lnot v}$, $P^\prime _{\lnot v}$ and $P^{\prime \prime }_{\lnot v}$ in *M*, $M^\prime$ and $M^{\prime \prime }$ (respectively) with respect to *u*. Further, let $P_v$ and $Q_v$ be the paths complementary to $P_{\lnot v}$ and $Q_{\lnot v}$ (respectively) in *M* with respect to *v*, and note that $P_v$ and $Q_v$ are paths complementary in *M* with respect to *u*. We define $P^\prime _v$, $Q^\prime _v$, $P^{\prime \prime }_v$ and $Q^{\prime \prime }_v$ similarly, and note that they are also paths complementary with respect to *u* in $M^\prime$ and $M^{\prime \prime }$. We emphasise that all ‘*P*’ paths contain literal $\lnot u$, whereas all ‘*Q*’ paths contain literal *u*.

In order to show that $\text{ ref }(M,u)$ exhibits the independence of *x* on *v*, we need to prove that $P^{\prime \prime }_{\lnot v}[x] = P^{\prime \prime }_{v}[x]$ and $Q^{\prime \prime }_{\lnot v}[x] = Q^{\prime \prime }_{v}[x]$. (In contrast to the proof of Lemma 4, we cannot say that $\lnot u \in P$ is without loss of generality here. This is because we need to show that the reformed model exhibits the independence of *x* on *v*, not on the ‘reform variable’ *u*. Since the right reform $M^{\prime \prime }$ depends upon the state of the left reform $M^\prime$, the outcome for a pair of paths complementary with respect to *v* may in principle depend upon their polarity at *u*. Hence, we must show explicitly that the exhibited independence of *x* on *v* is preserved for paths containing $\lnot u$*and* for those containing *u*.) Observe that $Q_{\lnot v}$ and $Q^\prime _{\lnot v}$ are identical paths by definition of left reform since $u \in Q$, and that $P^{\prime \prime }_{\lnot v}$ and $P^\prime _{\lnot v}$ are identical paths by definition of right reform since $\lnot u \in P^\prime$; hence $Q^\prime _{\lnot v}[x] = Q_{\lnot v}[x]$ and $P^{\prime \prime }_{\lnot v}[x] = P^\prime _{\lnot v}[x]$. Further, assume w.l.o.g. that $P_{\lnot v}[x] = x$. We consider four cases.

1. Suppose that $P_{\lnot v}[x] = Q_{\lnot v}[x]$. Observe that this implies $P^\prime _{\lnot v}[x] = P_{\lnot v}[x]$ by definition of left reform and reformed path (independently of $\mathcal {C}_\varPhi$), and hence $P^\prime _{\lnot v}[x] = Q^\prime _{\lnot v}[x]$. A symmetrical argument then shows that $P^{\prime \prime }_{\lnot v}[x] = P^\prime _{\lnot v}[x]$ and $Q_{\lnot v}^{\prime \prime }[x] = Q^\prime _{\lnot v}[x]$. It follows that $P_{\lnot v}^{\prime \prime }[x] = P_{\lnot v}[x]$ and $Q_{\lnot v}^{\prime \prime }[x] = Q_{\lnot v}[x]$.
2. Suppose that $P_{\lnot v}[x] \ne Q_{\lnot v}[x]$ and $(u,x) \notin \mathcal {C}_\varPhi$. Observe that $P^\prime _{\lnot v}[x] = Q_{\lnot v}[x]$ by definition of left reform and reformed path, since $P_{\lnot v}[u] = \lnot u$, $Q_{\lnot v}[x] = \lnot x$, and $(u,x) \notin \mathcal {C}_\varPhi$. Hence, $P^\prime _{\lnot v}[x] = Q^\prime _{\lnot v}[x]$, and we deduce that $P_{\lnot v}^{\prime \prime }[x] = P^\prime _{\lnot v}[x]$ and $Q_{\lnot v}^{\prime \prime }[x] = Q^\prime _{\lnot v}[x]$, using the same argument as for case (1). We conclude that $P^{\prime \prime }_{\lnot v}[x] = Q^{\prime \prime }_{\lnot v}[x] = Q_{\lnot v}[x]$.
3. Suppose that $P_{\lnot v}[x] \ne Q_{\lnot v}[x]$, $(u,x) \in \mathcal {C}_\varPhi$ and $(\lnot u, \lnot x) \notin \mathcal {C}_\varPhi$. First, observe that $P^\prime _{\lnot v}[x] = P_{\lnot v}[x]$ by definition of left reform and reformed path, since $P_{\lnot v}[u] = \lnot u$, $Q_{\lnot v}[x] = \lnot x$, and $(u,x) \in \mathcal {C}_\varPhi$. Second, observe that $Q^{\prime \prime }_{\lnot v}[x] = P^\prime _{\lnot v}[x]$ by definition of right reform and reformed path, since $Q^\prime _{\lnot v}[u] = u$, $P^\prime _{\lnot v}[x] = x$, and $(\lnot u, \lnot x) \notin \mathcal {C}_\varPhi$. We conclude that $P^{\prime \prime }_{\lnot v}[x] = Q^{\prime \prime }_{\lnot v}[x] = P_{\lnot v}[x]$.
4. Suppose that $P_{\lnot v}[x] \ne Q_{\lnot v}[x]$, $(u,x) \in \mathcal {C}_\varPhi$ and $(\lnot u, \lnot x) \in \mathcal {C}_\varPhi$. Similarly as for case (3), observe that $P^\prime _{\lnot v}[x] = P_{\lnot v}[x]$. This implies that $P^\prime _{\lnot v}[x] \ne Q^\prime _{\lnot v}[x]$. From here, a symmetrical argument (utilising the assumption $(u,x) \in \mathcal {C}_\varPhi$) shows that $Q_{\lnot v}^{\prime \prime }[x] = Q^\prime _{\lnot v}[x]$. We conclude that $P^{\prime \prime }_{\lnot v}[x] = P_{\lnot v}[x]$ and $Q^{\prime \prime }_{\lnot v}[x] = Q_{\lnot v}[x]$.

In all four cases, the argument is completely symmetrical with respect to the polarity of *v*; that is, the argument goes through if each $\lnot v$ subscript is replaced by *v*. In combination with the two facts $P_{\lnot v}[x] = P_v[x]$ and $Q_{\lnot v}[x] = Q_v[x]$, which follow from the lemma statement (*M* exhibits the indepedence of *x* on *v*), we hence deduce that $P^{\prime \prime }_{\lnot v}[x] = P_v^{\prime \prime }[x]$ and $Q^{\prime \prime }_{\lnot v}[x] = Q_v^{\prime \prime }[x]$ in all four cases. The four cases are clearly exhaustive. $\square$

With these latter three lemmata, we may now give a short inductive proof of full exhibition.

#### Theorem 8

$\mathcal {D}^{\textsf {rrs}}$ is fully exhibited.

#### Proof

Let $\varPhi = {\forall u_1 \cdots \forall u_m \exists x_1(S_1) \cdots \exists x_n(S_n)}\cdot {\phi }$ be a true QBF, and let

$$\mathcal {D}^{\textsf {rrs}} (\varPhi ) = {\forall u_1 \cdots \forall u_m \exists x_1(S^\prime _1) \cdots \exists x_n(S^\prime _n)}\cdot {\phi }.$$

Further, let $M_0$ be a model for $\varPhi$ and, for each $i \in [m]$, let $M_i = \text{ ref }(M_{i-1},u_i)$.

By induction on $i \in [m]$, we prove that $M_i$ is a model for $\varPhi$ that, for each $1 \le k \le i$ and for each $j \in [n]$ for which $u_k \notin S^\prime _j$, exhibits the independence of $x_j$ on $u_k$. Hence at step $i=m$ we prove that $M_m$ is a model for $\mathcal {D}^{\textsf {rrs}} (\varPhi )$. It follows that $\mathcal {D}^{\textsf {rrs}} (\varPhi )$ is a true DQBF, and that $\mathcal {D}^{\textsf {rrs}}$ is fully exhibited.

For the base case $i=1$, observe that $M_1 = \text{ ref }(M,u_1)$ is model for $\varPhi$ by Lemma 3, and that $M_1$ exhibits the independence of $u_1$ on $x_j$ for each $j \in [n]$ with $u_1 \notin S^\prime _j$, by Lemma 4. For the inductive step, let $i \in [m]$ and, suppose that $M_{i-1}$ is a model for $\varPhi$ that, for each $1 \le k \le i-1$ and for each $j \in [n]$ for which $u_k \notin S^\prime _j$, exhibits the independence of $x_j$ on $u_k$. Then $M_i = \text{ ref }(M_{i-1},u_i)$ is a model for $\varPhi$ that exhibits the independence of $u_i$ on $x_j$, for each $j \in [n]$ with $u_i \notin S^\prime _j$, by Lemmas 3 and 4. Also, by Lemma 5, the independences exhibited by $M_{i-1}$ are also exhibited by $M_i$. It follows that $M_i$ is a model for $\varPhi$ that, for each $1 \le k \le i$ and for each $j \in [n]$ for which $u_k \notin S^\prime _j$, exhibits the independence of $x_j$ on $u_k$. This completes the inductive step, and the proof. $\square$

We conclude this section by stating the following corollary. It is the immediate consequence of the fact that full exhibition is sufficient for soundness in all four dependency scheme systems (Theorems 2 and 7), combined with the full exhibition of $\mathcal {D}^{\textsf {rrs}}$ (Theorem 8). Note that the soundness results carry over to the weaker scheme $\mathcal {D}^{\textsf {std}}$, since $\textsf {P}(\mathcal {D}^{\textsf {rrs}})$ always simulates $\textsf {P}(\mathcal {D}^{\textsf {std}})$.

#### Corollary 2

Both $$\textsf {P}(\mathcal {D}^{\textsf {rrs}})$$ and $$\textsf {P}(\mathcal {D}^{\textsf {std}})$$ are sound, where $$\textsf {P}$$ is any of the calculi $$\forall $$$$\textsf {Exp+Res}$$, $$\textsf {IR-calc}$$, $$\textsf {Q-Res}$$ and $$\textsf {QU-Res}$$.

## Conclusions and Open Problems

We proposed an approach which strengthens the relationship between DQBF and QBF dependency schemes, furnishing fruitful connections between the two fields. By placing full exhibition at the centre of investigation, we have developed a method that gives rise to soundness results across calculi, a significant improvement over some existing ad hoc (and complex) proofs of soundness in the literature (cf. [37]). Theorems 4 and 5 highlight the importance of expansion-based proof systems for DQBF. Theorem 7 demonstrates that fully exhibited dependency schemes can be incorporated into expansion-based solving, as suggested in [22]. Theorem 8 proves Slivovsky’s conjecture that $\mathcal {D}^{\textsf {rrs}}$ is fully exhibited [35]; therefore either of $\mathcal {D}^{\textsf {rrs}}$ and $\mathcal {D}^{\textsf {std}}$ may be implemented in expansion solving (Corollary 2).

The relative proof complexities of $\textsf {Q-Res}$ and $\textsf {Q}(\mathcal {D})\textsf {-Res}$ were recently investigated, showing an exponential separation when $\mathcal {D}$ is $\mathcal {D}^{\textsf {rrs}}$ [11]. The proof complexities of expansion-based systems parametrised by dependency schemes remains open, but we conjecture the same exponential separation between $\textsf {IR-calc}$ and $\textsf {IR}(\mathcal {D}^{\textsf {rrs}})\textsf {-calc}$. We showed that the extent of incompleteness in $\textsf {QU-Res}$ can be rephrased in terms of the non-fully-exhibited schemes $\mathcal {D}$ for which $\textsf {Q}(\mathcal {D})\textsf {-Res}$ is sound. The exact extent remains open; that is, it is an open problem to characterise exactly the false DQBFs for which there is no $\textsf {DQBF-Q-Res}$ refutation.

## Footnotes

1 The concept originates from [35]; the term ‘full exhibition’ was coined in [6].

2 It is more conventional to write a QBF with a linearly ordered prefix, but it is convenient in this work to maintain the same notation for QBFs and DQBFs.

3 We note that the definition from [31] includes additional conditions, but we choose to omit them here, as they do not feature in this work.

4 Note that the fully exhibited scheme is not necessary polynomial-time computable.

5 Negative universal literals appear in the left-hand nodes in the conventional depiction of an assignment tree.
